# Identification of stable heat tolerance QTLs using inter-specific recombinant inbred line population derived from GPF 2 and ILWC 292

**DOI:** 10.1371/journal.pone.0254957

**Published:** 2021-08-09

**Authors:** Ashutosh Kushwah, Dharminder Bhatia, Inderjit Singh, Mahendar Thudi, Gurpreet Singh, Shayla Bindra, Suruchi Vij, B. S. Gill, Chellapilla Bharadwaj, Sarvjeet Singh, Rajeev K. Varshney

**Affiliations:** 1 Department of Plant Breeding and Genetics, Punjab Agricultural University, Ludhiana, Punjab, India; 2 Center of Excellence in Genomics and Systems Biology (CEGSB), International Crops Research Institute for the Semi-Arid Tropics (ICRISAT), Patancheru, India; 3 Regional Research Station, Faridkot, Punjab Agricultural University, Ludhiana, Punjab, India; 4 Division of Genetics, ICAR-Indian Institute of Agricultural Research (IARI), New Delhi, India; 5 State Agricultural Biotechnology Centre, Centre for Crop and Food Innovation, Food Futures Institute, Murdoch University, Murdoch, Western Australia, Australia; National Institute for Plant Genome Research, INDIA

## Abstract

Heat stress during reproductive stages has been leading to significant yield losses in chickpea (*Cicer arietinum* L.). With an aim of identifying the genomic regions or QTLs responsible for heat tolerance, 187 F_8_ recombinant inbred lines (RILs) derived from the cross GPF 2 (heat tolerant) × ILWC 292 (heat sensitive) were evaluated under late-sown irrigated (January-May) and timely-sown irrigated environments (November-April) at Ludhiana and Faridkot in Punjab, India for 13 heat tolerance related traits. The pooled ANOVA for both locations for the traits namely days to germination (DG), days to flowering initiation (DFI), days to 50% flowering (DFF), days to 100% flowering (DHF), plant height (PH), pods per plant (NPP), biomass (BIO), grain yield (YLD), 100-seed weight (HSW), harvest index (HI), membrane permeability index (MPI), relative leaf water content (RLWC) and pollen viability (PV)) showed a highly significant difference in RILs. The phenotyping data coupled with the genetic map comprising of 1365 ddRAD-Seq based SNP markers were used for identifying the QTLs for heat tolerance. Composite interval mapping provided a total of 28 and 23 QTLs, respectively at Ludhiana and Faridkot locations. Of these, 13 consensus QTLs for DG, DFI, DFF, DHF, PH, YLD, and MPI have been identified at both locations. Four QTL clusters containing QTLs for multiple traits were identified on the same genomic region at both locations. Stable QTLs for days to flowering can be one of the major factors for providing heat tolerance as early flowering has an advantage of more seed setting due to a comparatively longer reproductive period. Identified QTLs can be used in genomics-assisted breeding to develop heat stress-tolerant high yielding chickpea cultivars.

## Introduction

Chickpea (*Cicer arietinum* L.) or Garbanzo beans is a cool season food legume, originated from South-Eastern Turkey [1]. It is the second most consumed grain legume after dry bean grown worldwide. It is a self-pollinated diploid (2*n* = 2*x* = 16) crop with genome size of 738 Mb [2]. Chickpea is a nutrient-rich legume crop that contains 17–31% protein and significant amount of essential amino acids, vitamins and minerals [3]. It is free from anti-nutritional factors thereby the consumer preference for this legume is increasing. Despite of its economic importance, neither the area under cultivation nor productivity has increased to a desired level to meet the current demands. This sluggish pace of production trend is due to several abiotic and biotic constraints that have been challenging the crop [4,5]. Among abiotic stresses, heat stress is considered as one of the major constraints that affects the chickpea production. Chickpea is grown in winter season (November to April) in Northern India and experiences a high temperature (>35°C) stress during the reproductive phase (mid-February to April). Studies on the impact of climate change on chickpea production underlined the effect of warmer temperatures on crop development and the yield. For example, rise in temperature of 1°C reduces the chickpea yield up to 301 kg/ha in India [6]. The comparatively narrow genetic base of chickpea makes it vulnerable to high temperature which has a detrimental effect on its production [7]. Thus, there is a vital call for developing chickpea varieties that are heat resilient.

The effects of heat stress during the vegetative and reproductive growth stages using agronomic, morphological, phenological and physiological parameters have been studied in major crops such as wheat [8]; rice [9] and cotton [10], while limited studies have been conducted in chickpea [11]. In several studies, reproductive stage of the crop plant has been observed as the most sensitive stage of plant to heat stress [12]. Pod formation and seed set are adversely affected in chickpea if temperature rises above the threshold level, leading to reduction in grain yield [11,13]. Tissue hydration, crucial for physiological processes, measured by relative leaf water contents (RLWCs), is reduced during seedling, early flowering and pod development stages in chickpea subjected to stress [14]. Severe heat stress raises the temperature at cellular level, causes damage to the cell walls and increases the electrolyte leakage [15,16] thus, serve as an important adaptation to carry signals for induction of programmed cell death and assists to assimilate remobilization for development of seeds [16].

Heat stress tolerance is a complex trait and thus, an effective and simple screening method having well-defined traits for selecting heat-tolerant genotypes under field conditions is indispensable [17]. Genotype by environment (G × E) interaction also hampers the direct selection of heat-tolerant genotypes. Visual inspection, selection for physiological attributes related to plant response to high temperature, empirical selection for yield and marker assisted selection (MAS) are the four important selection methods which are used to improve heat tolerance through breeding [18]. Due to instability and poor heritability, lower genotypic variance for seed yield under stress [19], quantitative nature of traits, prevalence of linkage between desired and undesired genes [20] and complex genetic background of traits [21], breeding for yield under heat stress condition by means of conventional approaches has not been fairly successful over the years. Under such circumstances, molecular breeding seems to be a better strategy that can be deployed by targeting heat tolerance related traits.

In chickpea until 2005, about 150 SSR markers and sparse genetic maps were available which have limited efficacy for trait dissection. During last decade, chickpea research community has decoded the chickpea genome [2,22] and developed several genomic [23–25] and transcriptomic resources [26,27] that have transformed chickpea from “orphan legume crop” to “genomics resource rich legume crop” [28]. Now, several high-density genetic maps, physical maps and consensus maps are available for trait dissection [29–34] which provide new opportunities for accelerating research for faster genetic gains in chickpea. With the rapid development in next generation sequencing technologies, accelerated genotyping platforms such as genotyping by sequencing (GBS) has become a widely used approach in genetic studies. Backed by power of NGS, GBS is a high throughput and low cost genotyping method that can mine thousands of SNPs across the genome in number of individuals in a mapping population in very less time [35]. Restriction-site-associated DNA sequencing (RAD-seq) has been effectively applied for development of SNP markers, high-density genetic map construction, QTL mapping, phylogenetic research and population genetics [36]. Double Digestion Restriction-site-Associated DNA sequencing (ddRAD-seq) technique, developed [37], can adjust number of fragments by utilizing two different restriction enzymes [38] and exclusively uses size selection for recovering the appropriate number of regions, which arbitrarily distributed throughout the genome and maximizes the ability of multiplexing of numerous samples [39].

Chickpea is known to have narrow genetic base as compared to most other legumes [40,41]. Due to relatively low levels of polymorphism, inter-specific crosses between *C*. *arietinum* and *C*. *reticulatum* have been the primary focus for genetic studies [42]. The amount of polymorphism in an inter-specific mapping population varied from 16% to 36%, whereas 9.5% only in an intra-specific mapping population [23]. High-resolution genetic linkage maps can also be constructed by exploiting the inter-specific polymorphisms between *C*. *arietinum* and *C*. *reticulatum* [43]. Variation detection based on SNPs has also shown the similar trends. Thus, an inter-specific mapping population from a cross between GPF 2 (*C*. *arietinum*) and ILWC 292 (*C*. *reticulatum*) has been used in the present study to identify the key genomic regions of heat tolerance related traits using ddRAD-seq based genotyping and phenotyping in contrasting environmental conditions. Chickpea cultivar GPF2 is a semi erect, medium tall cultivar released by Punjab Agricultural University, Ludhiana, Punjab and recommended for cultivation in Punjab state and in North Western Plains Zone of India. Another parent of RILs, ILWC292 (*C*. *reticulatum*) is the wild species of chickpea having semi prostrate growth habit. After evaluating the RIL population under late-sown irrigated and timely-sown irrigated environments at two locations and generating ddRAD-Seq data, this study reports a genetic map for the above mentioned population and identification of QTLs associated with heat tolerance. Some of these QTLs, after validation, should be useful in genomics-assisted breeding for heat stress tolerance in chickpea.

## Materials and methods

### Plant materials and phenotyping

**Fig 1:**
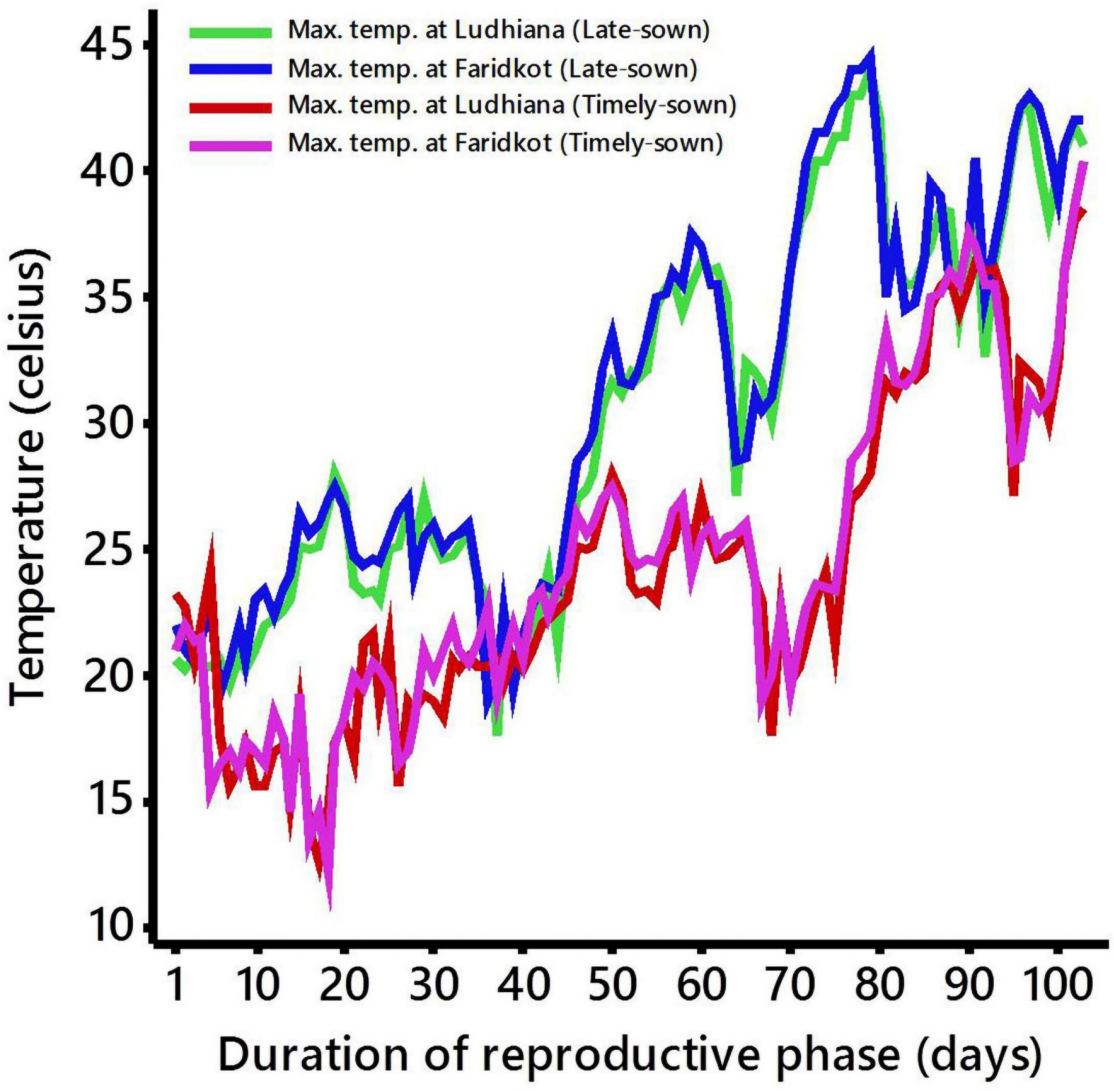
Daily maximum temperatures for late-sown as well as timely-sown conditions during the reproductive phase at both locations (Ludhiana and Faridkot).

A total of 187 recombinant inbred lines (RILs) segregating for heat tolerance related traits from an inter-specific cross of cultivar GPF 2 (heat tolerant) × *C*. *reticulatum* acc ILWC 292 (heat sensitive) developed using single seed descent method. The RIL population along with parents was planted during winter’s season of 2017–18 in alpha lattice design (17 × 12) under timely-sown (November-April) and late-sown (January-May) conditions with three replications at two locations, i.e., Ludhiana and Faridkot. The Ludhiana (30.9010° N, 75.8573° E) and Faridkot (30.6769° N, 74.7583° E) sites are categorized as a semi-arid sub-tropical region and semi-arid dry region, respectively. Both sites comprise loamy sand with 59.8% sand and 16.5% clay (Typic Ustorthents). The average annual rainfall is 700 mm at Ludhiana and 450 mm at Faridkot, of which more than 70% occurs from July to September. Each RIL was sown in paired rows of 2 m length at 30 cm × 10 cm spacing. The late-sown chickpea was exposed to terminal heat stress because the conserved soil moisture recedes as the season progresses and the temperature rises [44,45]. Thus, heat tolerance related traits have been studied in late-sown irrigated condition, using the timely-sown irrigated condition as a control. During the screening of heat tolerance, irrigation was provided to avoid the confounding effect of drought stress. The daily maximum temperatures for late-sown as well as timely-sown conditions during the reproductive phase at both the locations (Ludhiana and Faridkot) were recorded (Fig 1).

Phenotypic data were collected for a total of 13 heat tolerance related traits *viz*., days to germination (DG), days to flowering initiation (DFI), days to 50% flowering (DFF), days to 100% flowering (DHF), plant height (PH), number of pods per plant (NPP), biomass (BIO), yield (YLD), 100-seed weight (HSW), harvest index (HI), membrane permeability index (MPI), relative leaf water content (RLWC) and pollen viability (PV). Randomly five plants were selected to record the observations on PH, NPP, BIO and YLD in each plot. The data on DG, DFI, DFF, DHF and HSW were recorded on plot basis. HI was calculated as:

Harvest Index (HI) = (seed yield/total shoot biomass) ×100

Pollen viability test was studied by collecting the pollen samples at the time of 50% flowering. The pollen viability was observed by using 2% acetocarmine stain described by [46].

The MPI was determined according to the method described earlier [47] and modified [48] using following formula:

Membrane permeability index (MPI) = [1- (C1/C2)] ×100

Where, C1 = Initial electrical conductivity at (40°C); C2 = Final electrical conductivity at (100°C).

The RLWC was calculated by the formula [49] using following formula:

RLWC (%) = (FW-DW / TW-DW) ×100

Where, FW = Fresh weight, DW = Dry weight, TW = Turgid weight.

### Analysis of variance (ANOVA), best linear unbiased predictor(s) (BLUPs) and correlation coefficient analysis

The ANOVA was calculated for individual environments using mixed model analysis to estimate the contribution made by each factor to the total variation using SAS-software version 9.3 [50]. The data from timely-sown and late-sown conditions were used to estimate BLUPs using the residual maximum likelihood algorithm (ReML) in R package lmer [51]. BLUPs were estimated for 13 traits and scatter plots were drawn for all the traits using BLUPs to find the correlation between two locations i.e., Ludhiana and Faridkot.

### QTL analysis

The RIL population was genotyped with ddRAD-seq [37] that used restriction enzymes *PstI* and *MspI* (Thermo Fisher Scientific, MA, United States). The ddRAD-seq data analysis of RILs for SNP discovery and development of linkage map has already been described earlier [52]. The QTL analysis has been performed with the composite interval mapping (CIM) method executed in the Windows QTL Cartographer V2.5 software package [53] using genotypic and phenotypic data. The CIM analysis was run using forward and backward stepwise regression. For each trait, experiment-wise significance thresholds (*p =* ≤0.05) were determined with 1000 permutations for QTL detection. The position of the QTLs was identified on the basis of its logarithm of odds (LOD) peak location with 95% confidence interval. The LOD score of 3 has been adapted as threshold LOD value. The percentage of phenotypic variance and additive effect described by QTLs was also estimated. The phenotypic contribution (R^2^) was estimated as the percentage of variance explained by each QTL in proportion to the total phenotypic variance, while additive effect was estimated to find the positive or negative effect for the respective trait.

## Results

### Phenotypic performance of the mapping population

**Fig 2:**
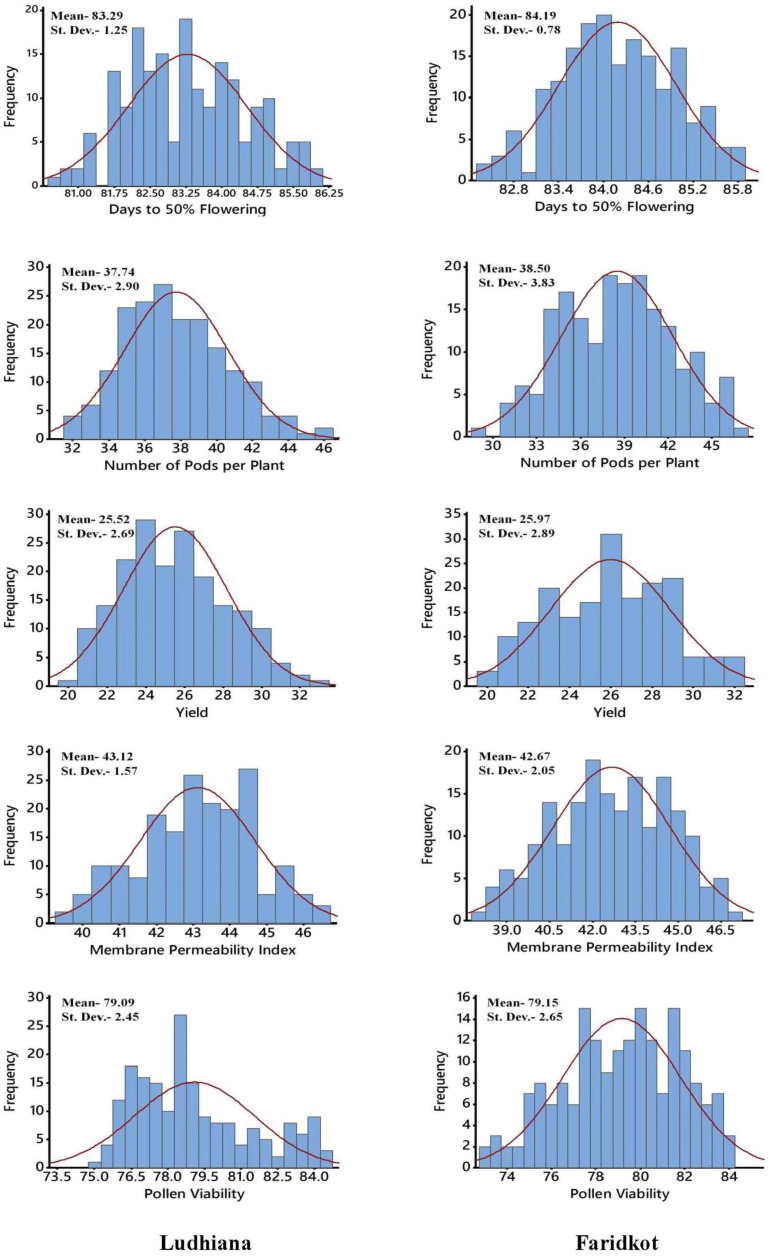
Graphical representations of RILs for the various traits in chickpea using pooled phenotypic data between timely-sown and late-sown condition at Ludhiana and Faridkot.

The RILs along with parents were evaluated in timely-sown (non-stress) and late-sown (heat-stress) conditions at Ludhiana and Faridkot. The late-sown condition in chickpea exposed the RIL population to heat stress during reproductive stage as the maximum temperature crossed the threshold limit during that period at both the locations (Fig 1). Significant variation was observed for heat stress related traits among the RILs as well as the parents under timely-sown and late-sown conditions (Table 1, Fig 2, S1 and S2 Figs). The contrast analysis of parents for all the traits depicts that there were highly significant differences between parents under timely-sown and late-sown conditions (Table 1). All the traits were significantly affected by heat stress environment, except HSW and HI which were moderately affected. The pooled ANOVA for both locations in timely-sown as well as late-sown conditions for all the traits showed highly significant differences in RILs for genotypic variance (Table 1). Significant differences were also observed for genotype × location (G × L) interaction variance for all the traits, except DG, DFI, DFF and DHF.

**Table 1:** Mean performance of RILs evaluated under timely-sown and late-sown conditions based on pooled data from Ludhiana and Faridkot.

Trait	Environment	ILWC 292	GPF 2	Contrast between parents	Mean	Coefficient of variation (%)	Range	Genotypic variance	Genotype × location interaction variance
Days to germination	Timely sown	12.34	8.12	44.33**	9.33	5.05	8.12–12.34	1.50**	0.51
	Late sown	15.53	10.67	175.00**	13.12	9.23	10.63–16.16	5.09**	0.27
Days to flowering initiation	Timely sown	90.33	82.8	78.87**	85.99	2.01	78.91–90.33	1.96**	0.15
	Late sown	82.82	75.86	11.53*	75.42	4.15	69.17–83.49	6.01**	0.42
Days to 50% flowering	Timely sown	94.26	86.46	171.50**	89.46	2.08	82.35–94.26	2.24**	0.14
	Late sown	84.74	78.76	19.17**	78.15	3.97	72.26–86.11	9.92**	0.36
Days to 100% flowering	Timely sown	98.08	89.78	171.50**	93.1	1.96	86.67–98.08	2.28**	0.2
	Late sown	88.27	82.66	10.77*	81.99	3.86	75.75–90.05	8.77**	0.59
Plant height (cm)	Timely sown	42.42	58.87	65.43**	45.68	10.47	33.82–58.87	4.05**	3.85**
	Late sown	21.82	43.9	125.85**	29.33	32.08	15.14–48.88	45.96**	5.11**
Number of pod per plant	Timely sown	43.53	68.54	133.10**	47.39	21.75	25.13–75.07	18.64**	5.82**
	Late sown	20.3	43.96	117.41**	29.26	31.29	14.65–48.28	35.46**	6.04**
Biomass per plant (g)	Timely sown	76.78	113.32	55.17**	81.33	15.47	51.55–113.70	10.35**	4.12**
	Late sown	38	76.26	131.55**	57.03	30.29	30.16–90.32	64.94**	7.93**
Yield per plant (g)	Timely sown	27.91	49.74	232.07**	32.14	27.07	14.13–54.69	18.16**	6.12**
	Late sown	12.35	33.39	125.87**	19.52	43.33	8.35–38.07	53.39**	5.15**
100 seed weight (g)	Timely sown	11.27	16.18	1629.73**	14.22	16.11	9.79–18.42	20.41**	18.35**
	Late sown	9.32	15.85	174.08**	13.06	22.24	7.92–18.20	46.74**	5.61**
Harvest index (%),	Timely sown	36.63	43.55	15.81**	38.98	16.48	22.49–52.86	12.52**	5.31**
	Late sown	32.61	43.97	22.90**	32.85	16.1	21.75–44.87	19.04**	2.45**
Membrane permeability index	Timely sown	42.23	28.81	150.71**	39.42	12.12	28.70–50.76	15.49**	8.51**
	Late sown	52.71	38.03	12.71**	46.28	15.82	32.23–59.71	27.38**	3.70**
Relative leaf water content (%)	Timely sown	65.28	88.31	178.91**	74.85	9.37	59.06–89.94	6.07**	10.17**
	Late sown	47.72	78.61	290.43**	62.41	17.05	45.96–79.89	29.92**	5.53**
Pollen viability (%)	Timely sown	81.29	93.09	27.36**	84.74	7.4	70.99–94.77	10.83**	5.10**
	Late sown	69.43	85.34	36.61**	73.55	13.59	58.98–93.13	22.49**	5.37**

* = Significant at 5% probability level

** = Significant at 1% probability level.

### Correlation between locations

**Fig 3:**
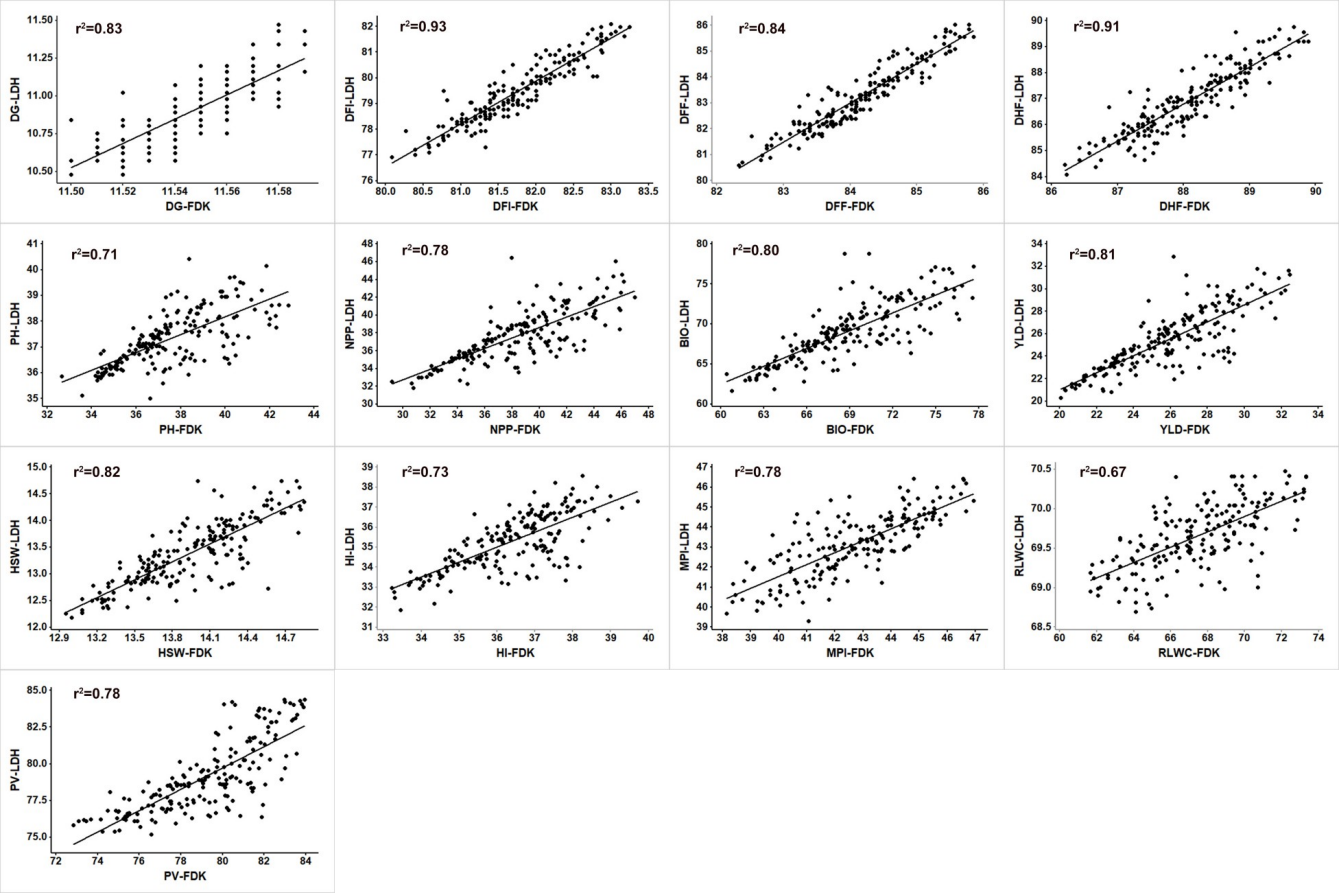
The scatter plots of heat stress related traits showing relationship between both locations, i.e., Ludhiana and Faridkot. The straight was plotted as regression line. DG = days to germination, DFI = days to flowering initiation, DFF = days to 50% flowering, DHF = days to 100% flowering, PH = plant height (cm), NPP = Number of pod per plant, BIO = biomass per plant (g), YLD = yield per plant (g), HSW = 100-seed weight (g), HI = harvest index (%), MPI = membrane permeability index, RLWC = relative leaf water content (%), PV = pollen viability (%), LDH = Ludhiana location, FDK = Faridkot location.

To identify the QTLs that could be consistent at two different locations, BLUPs (Best Linear Unbiased Predictors) for genotypes were identified for both the locations (S1 Table). Even though there was significant G × L interaction, the scatter plots using BLUP values showed highly significant relationship between locations for most of the traits except PH, HI and RLWC which showed moderately high correlation coefficient (Fig 3). Correlation coefficient (r^2^) ranged from 0.67 for RLWC to 0.94 for DFF between the two locations.

### QTLs identified for heat-stress tolerance

**Fig 4:**
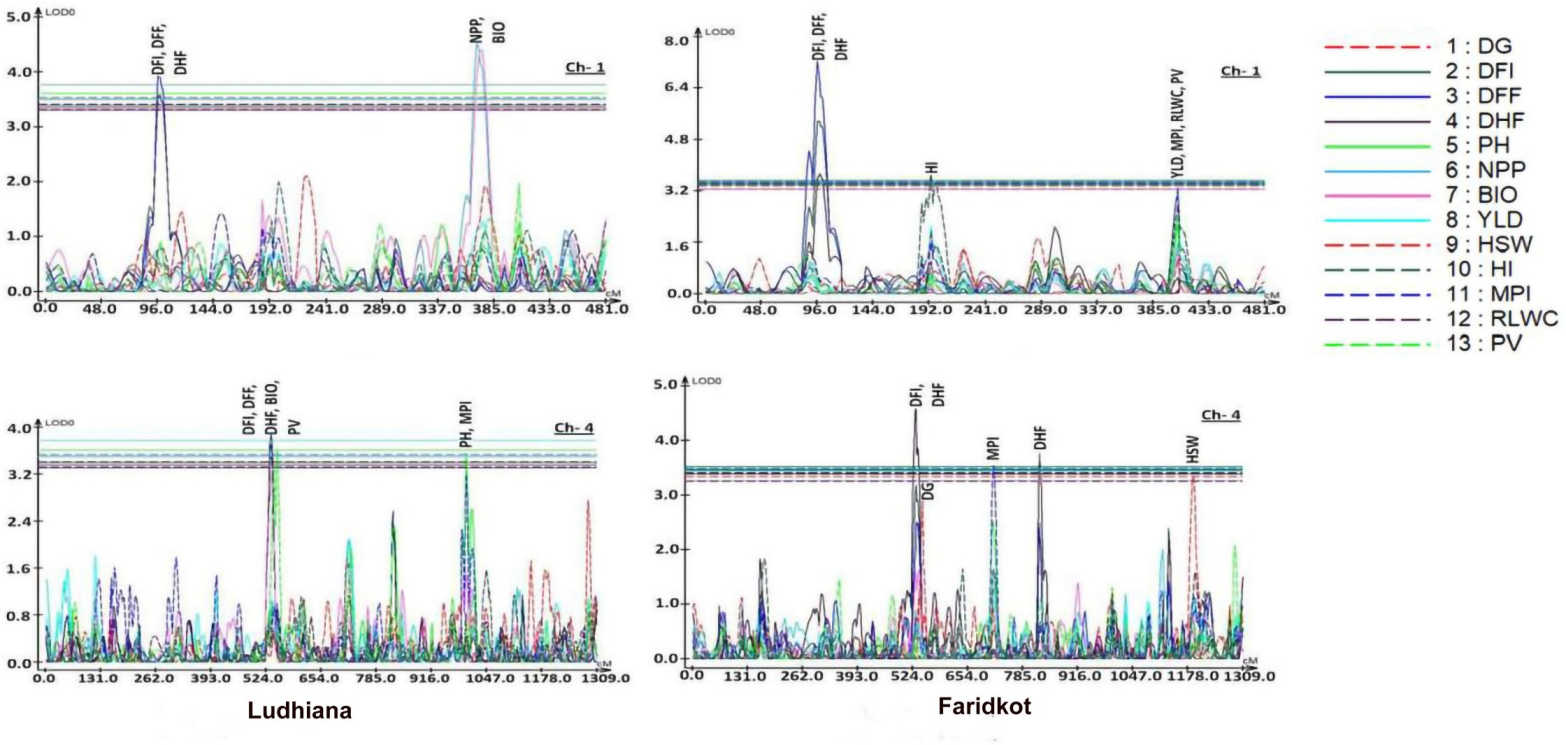
Logarithm of odds ratio (LOD) curves obtained by composite interval mapping for quantitative trait loci (QTLs) mapped for different traits in RIL population (GPF 2 × *C*. *reticulatum* acc ILWC 292) in heat stress environment.

Generation of genotyping data for 1365 filtered and parental polymorphic SNPs and construction of linkage map has been described earlier [52]. Here, genotypic data of 1365 informative SNPs, linkage map distances and BLUP values of two different locations were used to identify QTLs for heat stress tolerance related traits. A total of 28 QTLs for Ludhiana and 23 QTLs at Faridkot were identified for 13 different traits (Tables 2 and 3; Fig 4, S3 Fig). Out of these, 13 stable QTLs for DG, DFI, DFF, DHF, PH, YLD and MPI have been identified at both the locations (highlighted with bold in Tables 2 and 3). Four QTL clusters containing QTLs for DG, DFI, DFF, DHF, PH and MPI were identified on chromosome 1, 2, 4 and 6 on the same genomic position at both the locations (Fig 4, S2 Fig). All of these QTLs were distributed on seven linkage groups, while linkage group on chromosome 8 harbours no QTL. Maximum QTLs were present on chromosomes 1 and 4 at both the locations. The highest phenotypic variation was observed for biomass (13.71%) at Ludhiana and days to 50% flowering (18.30%) at Faridkot. The highest LOD value was observed for days to germination (5.27) at Ludhiana and days to 50% flowering (7.26) at Faridkot. QTLs having positive or negative additive effect for a particular trait imply that the increase in the proportion of the phenotypic variation of that particular trait is contributed by the allele from GPF 2 or *C*. *reticulatum* acc ILWC 292, respectively.

**Table 2:** Summary of the QTLs associated with the heat stress related traits at Ludhiana.

Trait	Chr.	QTL name	LOD	Additive	Phenotypic variation explained(%)	Left flanking marker	Right flanking marker
				effect			
Days to germination	5	*qdg-01*	3.53	0.0828	8.98	CNC_021164.1.40739029	CNC_021164.1.39765508
	**6**	***qdg-02***	**5.27**	**0.0985**	**11.3**	**CNC_021165.1.18056125**	**CNC_021165.1.513774**
	**7**	***qdg-03***	***2*.*3***	***0*.*0529***	***5*.*59***	**CNC_021166.1.28474588**	**CNC_021166.1.8118822**
Days to flowering initiation	**1**	***qdfi-01***	***3*.*59***	***0*.*5876***	***9*.*21***	**CNC_021160.1.35885685**	**CNC_021160.1.8023246**
	**4**	***qdfi-02***	***3*.*88***	***-0*.*4694***	***9*.*94***	**CNC_021163.1.11351378**	**CNC_021163.1.11351447**
Days to 50% flowering	**1**	***qdff-01***	***3*.*93***	***0*.*6375***	***10*.*13***	**CNC_021160.1.35885685**	**CNC_021160.1.8023246**
	3	*qdff-02*	3.29	-0.4136	7.9	CNC_021162.1.21073044	CNC_021162.1.39357194
	**4**	***qdff-03***	***3*.*71***	***-0*.*4681***	***9*.*25***	**CNC_021163.1.11351378**	**CNC_021163.1.11351447**
	**6**	***qdff-04***	***3*.*07***	***0*.*469***	***6*.*8***	**CNC_021165.1.18056125**	**CNC_021165.1.513774**
Days to 100% flowering	**1**	***qdhf-01***	***3*.*58***	***0*.*5381***	***9***	**CNC_021160.1.35885685**	**CNC_021160.1.8023246**
	3	*qdhf-02*	3.11	-0.4143	7.6	CNC_021162.1.21073044	CNC_021162.1.39357194
	**4**	***qdhf-03***	***3*.*52***	***-0*.*4674***	***8*.*72***	**CNC_021163.1.13462111**	**CNC_021163.1.11351378**
	**4**	***qdhf-04***	***2*.*5***	***-0*.*412***	***5*.*7***	**CNC_021163.1.48163277**	**CNC_021163.1.48245021**
Plant height (cm)	**2**	***qph-01***	***3*.*66***	***-0*.*431***	***7*.*93***	**CNC_021161.1.31430073**	**CNC_021161.1.9956999**
	4	*qph-02*	3.56	0.5285	10.2	CNC_021163.1.38540774	CNC_021163.1.38370939
Number of pod per plant	1	*qnpp-01*	4.53	-1.4312	12.76	CNC_021160.1.41430352	CNC_021160.1.41044856
	3	*qnpp-02*	4.02	1.3582	11.77	CNC_021162.1.38774358	CNC_021162.1.29358942
Biomass per plant (g)	1	*qbio-01*	4.41	-1.6353	12.22	CNC_021160.1.41430352	CNC_021160.1.41044856
	4	*qbio-02*	3.33	1.642	13.71	CNC_021163.1.11351378	CNC_021163.1.11351447
Yield per plant (g)	**2**	***qyld-01***	***3*.*84***	***-1*.*1401***	***10*.*28***	**CNC_021161.1.31430073**	**CNC_021161.1.9956999**
	3	*qyld-02*	3.1	1.0001	8.6	CNC_021162.1.38774358	CNC_021162.1.29358942
100 seed weight (g)	6	*qhsw-02*	3.38	-0.2103	8.56	CNC_021165.1.41420023	CNC_021165.1.41646826
Membrane permeability index	**2**	***qmpi-01***	***2*.*52***	***0*.*5386***	***6*.*69***	**CNC_021161.1.3663690**	**CNC_021161.1.31430073**
	4	*qmpi-02*	3.21	-0.8137	10.07	CNC_021163.1.38540774	CNC_021163.1.38370939
Relative leaf water content (%)	7	*qrlwc-01*	3.34	0.158	9.15	CNC_021166.1.25250619	CNC_021166.1.42984094
Pollen viability (%)	2	*qpv-01*	4.54	-1.151	11.28	CNC_021161.1.3663690	CNC_021161.1.31430073
	4	*qpv-02*	3.33	1.2303	13.59	CNC_021163.1.11351447	CNC_021163.1.12812015
	4	*qpv-03*	3.61	0.9206	8.92	CNC_021163.1.12812016	CNC_021163.1.12811935

**Table 3:** Summary of the QTLs associated with the heat stress related traits at Faridkot.

Trait	Chr.	QTL name	LOD	Additive effect	Phenotypic variation explained (%)	Left flanking marker	Right flanking marker
Days to germination	**7**	***qdg-01***	***4*.*20***	**0.0067**	**8.9**	**CNC_021166.1.28474588**	**CNC_021166.1.8118822**
	**6**	***qdg-02***	***2*.*93***	**0.0069**	**6.7**	**CNC_021165.1.18056125**	**CNC_021165.1.513774**
Days to flowering initiation	**1**	***qdfi-01***	***5*.*39***	**0.3836**	**13.68**	**CNC_021160.1.35885685**	**CNC_021160.1.8023246**
	**4**	***qdfi-02***	***3*.*19***	**-0.2153**	**7.64**	**CNC_021163.1.11351378**	**CNC_021163.1.11351447**
Days to 50% flowering	1	*qdff-01*	4.46	0.4609	14.72	CNC_021160.1.33682907	CNC_021160.1.35885685
	**1**	***qdff-02***	***7*.*26***	**0.5151**	**18.3**	**CNC_021160.1.35885685**	**CNC_021160.1.8023246**
	**4**	***qdff-03***	***2*.*50***	**-0.2456**	**6.49**	**CNC_021163.1.11351378**	**CNC_021163.1.11351447**
	**6**	***qdff-04***	***3*.*09***	**0.2894**	**7.07**	**CNC_021165.1.18056125**	**CNC_021165.1.513774**
Days to 100% flowering	**1**	***qdhf-01***	***3*.*75***	**0.3482**	**9.06**	**CNC_021160.1.35885685**	**CNC_021160.1.8023246**
	**4**	***qdhf-02***	***4*.*58***	**-0.2849**	**9.94**	**CNC_021163.1.13462111**	**CNC_021163.1.11351378**
	**4**	***qdhf-03***	***3*.*76***	***-0*.*3236***	***8*.*07***	**CNC_021163.1.48163277**	**CNC_021163.1.48245021**
Plant height (cm)	**2**	***qph-01***	***2*.*28***	***-0*.*7371***	***6*.*19***	**CNC_021161.1.31430073**	**CNC_021161.1.9956999**
Yield per plant (g)	1	*qyld-01*	3.22	-1.193	7.86	CNC_021160.1.18699653	CNC_021160.1.30785636
	**2**	***qyld-02***	***2*.*48***	***-0*.*9072***	***6*.*87***	**CNC_021161.1.31430073**	**CNC_021161.1.9956999**
100 seed weight (g)	4	*qhsw-01*	*3*.*35*	*-0*.*1654*	*8*.*9*	CNC_021163.1.44961556	CNC_021163.1.44507694
	5	*qhsw-02*	3.73	0.178	11.11	CNC_021164.1.33290198	CNC_021164.1.19722937
HI	1	*qhi-01*	3.72	0.4889	7.82	CNC_021160.1.35223851	CNC_021160.1.35250808
	1	*qhi-02*	*3*.*07*	*0*.*4633*	*7*.*58*	CNC_021160.1.35250799	CNC_021160.1.35301203
	1	*qhi-03*	3.09	-0.5371	8.36	CNC_021160.1.18699653	CNC_021160.1.30785636
Membrane permeability index	1	*qmpi-01*	3.29	0.8733	8.04	CNC_021160.1.18699653	CNC_021160.1.30785636
	**2**	***qmpi-02***	***2*.*52***	***0*.*7048***	***7*.*81***	**CNC_021161.1.3663690**	**CNC_021161.1.31430073**
	4	*qmpi-03*	3.55	0.8254	9.23	CNC_021163.1.41223431	CNC_021163.1.44127944
Pollen viability (%)	5	*qpv-01*	3.46	-1.1695	12.16	CNC_021164.1.9654616	CNC_021164.1.47240495

## Discussion

Heat stress is increasingly becoming a severe constraint to chickpea production due to the changing scenario of chickpea cultivation and expected overall increase in global temperatures due to climate change. A threshold temperature of 35°C was found to be critical in differentiating heat tolerant and heat sensitive genotypes in chickpea under field conditions [44]. Chickpea suffers heavy yield losses when exposed to heat stress at the reproductive stage. In this study, late-sown condition was proved to be an ideal condition for heat tolerance screening as the temperature at the time of pod setting crossed the threshold limit (Fig 1). Late sowing exposes the chickpea to terminal heat stress condition as the season progresses; temperature increases and the conserved moisture depleted from the soil [45]. Heat stress during pod development reduced the seed yield at higher rate as compared to heat stress during early flowering [11]. However, earliness is the most significant trait offering tolerance to heat and drought stress. Thus, late sowing is effective for heat tolerance screening in chickpea [54].

Interspecific RIL population and its parents showed significant differences for yield and yield contributing traits and physiological traits in late-sown as compared to timely-sown condition. Most of the morphological and physiological traits were significantly affected by heat stress environment in some previous studies [44,55–58]. Overall, there was reduction in seed yield in RILs under heat stress conditions. Low pollen viability in the RILs could be one of the major causes of reduced seed yield during heat stress environment [59]. Pollen sterility was reported to be one of the major reasons for poor pod setting during pre-anthesis high temperature stress [60]. Low pollen viability, indehiscent anthers and other anther abnormalities are associated with poor pod set during pre-anthesis high temperature stress [61]. Whereas, high temperature stress during post-anthesis is related with poor pollen germination, pollen tube growth and fertilization [62,63]. Development of male and female reproductive parts like pollen and stigma are the most sensitive organs to heat stress in reproductive biology [64]. Pod set percentage was reduced at high temperatures in chickpea and concluded that pollen viability is the major reason of sterility under high temperature stress at anthesis in chickpea [57]. Thus, study of pollen grains may help to expect the genetic variations present among the genotypes for heat tolerance at reproductive phase. Late-sown condition adversely affected the physiological traits such as RLWC, MPI and morphological traits like plant height, total dry matter, grain yield and test weight as compared to controlled conditions [65]. Generally, reduced water availability is frequently associated with heat stress under field conditions [66]. The RLWC has been reduced due to increase in transpiration under heat stress condition [67,68]. Heat stress can reduce the grain yield by disturbing both source and sink relationship for photosynthate assimilates [17].

Variances due to G × L interaction were highly significant for all the traits except DG, DFI, DFF, DHF, which were non-significant at both locations. Both the locations had almost similar rise in temperature under late-sown condition with very little differences. Significant G × L interaction could be due to other factors than the temperature. To encounter these differences, BLUP values for genotypes for both the locations were also estimated taking location as random effect. BLUP values of RIL population for both the locations showed high correlations with each, thus showing that these can be used for further QTL analysis to find the consistent QTLs at both the locations. A highly significant genetic and genotype × environment interaction variance for pooled analysis of two heat stress environments for days to 50% flowering, pod setting percentage, biomass, number of filled pods, yield, harvest index, 100-seed weight and total number of seeds was reported [58]. Further, a highly significant genetic and genotype × environment interaction variance across the heat stress environments was also reported [69].

A little progress could be made to breed cultivars harbouring complex quantitative traits through conventional selection due to polygenic control and higher genotype x environment interaction [70]. Thus, mapping QTLs for complex quantitative traits is an important pre-requisite for understanding their genetic architecture and precise transfer in the background of commercial cultivars. A total of 28 QTLs at Ludhiana and 23 QTLs at Faridkot were identified for 13 traits in the RIL population evaluated under timely-sown and late-sown conditions. Out of these, 13 stable QTLs for DG, DFI, DFF, DHF, PH, YLD and MPI were identified at both the locations. The stable QTLs for DG have been reported first time in our study on chromosome 6 and 7. Though DG is not affected by heat stress under late-sown condition, but QTL represents the genotypic differences in RIL population for this trait, which can be used in marker assisted breeding programmes. A stable QTL for seed yield under heat stress conditions was identified on chromosome 2.

Early flowering has an advantage of more pod setting before the occurrence of heat stress due to comparatively longer reproductive phase. Thus, early flowering can be one of the major factors for providing tolerance against heat stress. The stable QTLs for flowering harbour on chromosome 1 and 4, suggesting that these loci confer flowering time in chickpea. The QTLs for seed yield were earlier reported [71] who identified three QTLs, while Rehman et al., [72] identified two QTLs located on chromosome 1. For seed yield, one QTL on chromosome 4 [73] and for seed weight, two QTLs on chromosome 4 and 8 [74] were mapped. Using GBS approach four QTLs for yield per plant located on chromosome 4, 6, 7 and 8 were reported [75]. Two QTLs for seed weight on chromosome 6 (LOD = 2.6) and 7 (LOD = 2.7) and two QTLs for plant height on chromosome 1 (LOD = 3.25) and 3 (LOD = 2.7) were identified [76]. QTL for 100-seed weight was identified [77] on chromosome 4 which was in accordance to our results. Several QTLs for plant height, number of pods per plant, 100-seed weight, biomass, harvest index and yield which were also at the same locus as identified in our study [31]. Likewise, several QTLs have been found [78] for plant height, number of pods per plant, 100-seed weight and yield which were at the same locus as identified in our study. More recently, 77 QTLs (37 major and 40 minor) were reported for 12 of 13 heat tolerance related traits, including a genomic region on CaLG07 harbours QTLs explaining >30% phenotypic variation for days to pod initiation, 100 seed weight [79]. Four QTL clusters containing QTLs for DG, DFI, DFF, DHF, PH and MPI identified on chromosome 1, 2, 4 and 6 on the same genomic position at both the locations. A total of nine QTL clusters for drought tolerance related traits identified [31], out of which one major cluster, present on chromosome 4, was referred as “*QTL-hotspot*”. Jaganathan et al. [33] refined this *“QTL-hotspot”* region by genotyping-by-sequencing (GBS) approach and identified 49 SNP markers in this region. Further, Kale et al. [34] partitioned this *“QTL-hotspot”* into two regions *“QTL-hotspot_a”* and *“QTL-hotspot_b”* and identified four promising candidate genes responsible for drought stress tolerance in chickpea. QTL clusters identified in our study can be targeted for marker-assisted breeding for introgression into elite cultivars to enhance heat stress tolerance.

QTLs for multiple traits identified at both the locations co-localised at same genomic position. These QTL regions could be prime target in breeding programme for improving chickpea cultivars under heat stress conditions. QTLs for multiple traits for single location were also identified, however no strong signals could be observed for other location. This could be due to significant variation observed for genotype × location. However, the present study has identified the potential genomic regions for important agronomic and physiological traits that could be used in further breeding programme. These identified QTLs will serve as a potential tool for identification of candidate genes with the recent advances in genomics and transcriptomics resources in chickpea.

## Conclusions

This study illustrated the presence of significant differences in interspecific RIL population and its parents for yield and yield contributing traits and physiological traits in late-sown as compared to timely-sown condition. Reduction in seed yield during heat stress could be associated with low pollen viability in the RILs. A total of 28 QTLs at Ludhiana and 23 QTLs at Faridkot location were identified for 13 traits using SNP genotyping by ddRAD-Seq and BLUPs in the RIL population evaluated under timely-sown and late-sown conditions. Out of these, 13 stable QTLs for 7 traits were identified at both the locations. The stable QTLs for days to germination have been reported first time in the present study. The stable QTLs for flowering suggesting that these loci confer flowering time in chickpea and early flowering has an advantage of more pod setting before the occurrence of heat stress due to comparatively longer reproductive phase. Four QTL clusters containing QTLs for multiple traits identified on the same genomic region at both locations which would be the prime target in breeding programme for improving heat stress tolerance in chickpea.

## Supporting information

S1 Fig Graphical representations of RILs for the various traits in chickpea using pooled phenotypic data between timely-sown and late-sown condition at Ludhiana. (TIF)

S2 Fig Graphical representations of RILs for the various traits in chickpea using pooled phenotypic data between timely-sown and late-sown condition at Faridkot. (TIF)

S3 Fig Logarithm of odds ratio (LOD) curves obtained by composite interval mapping for quantitative trait loci (QTLs) mapped for different traits in RIL population (GPF 2 × *C*. *reticulatum* acc ILWC 292) in heat stress environment. (JPG)

S1 Table Best linear unbiased prediction value (BLUPs) for RIL population of pooled phenotypic data between timely-sown and late-sown conditions at Ludhiana. (DOCX)

S2 Table Best linear unbiased prediction value (BLUPs) for RIL population of pooled phenotypic data between timely-sown and late-sown conditions at Faridkot. (DOCX)
