# Supplementary figures and images for: Identification of stable heat tolerance QTLs using inter-specific recombinant inbred line population derived from GPF 2 and ILWC 292
Source: PLoS One. 2021 Aug 9;16(8):e0254957. doi: 10.1371/journal.pone.0254957 (PMC8352073; doi:10.1371/journal.pone.0254957)

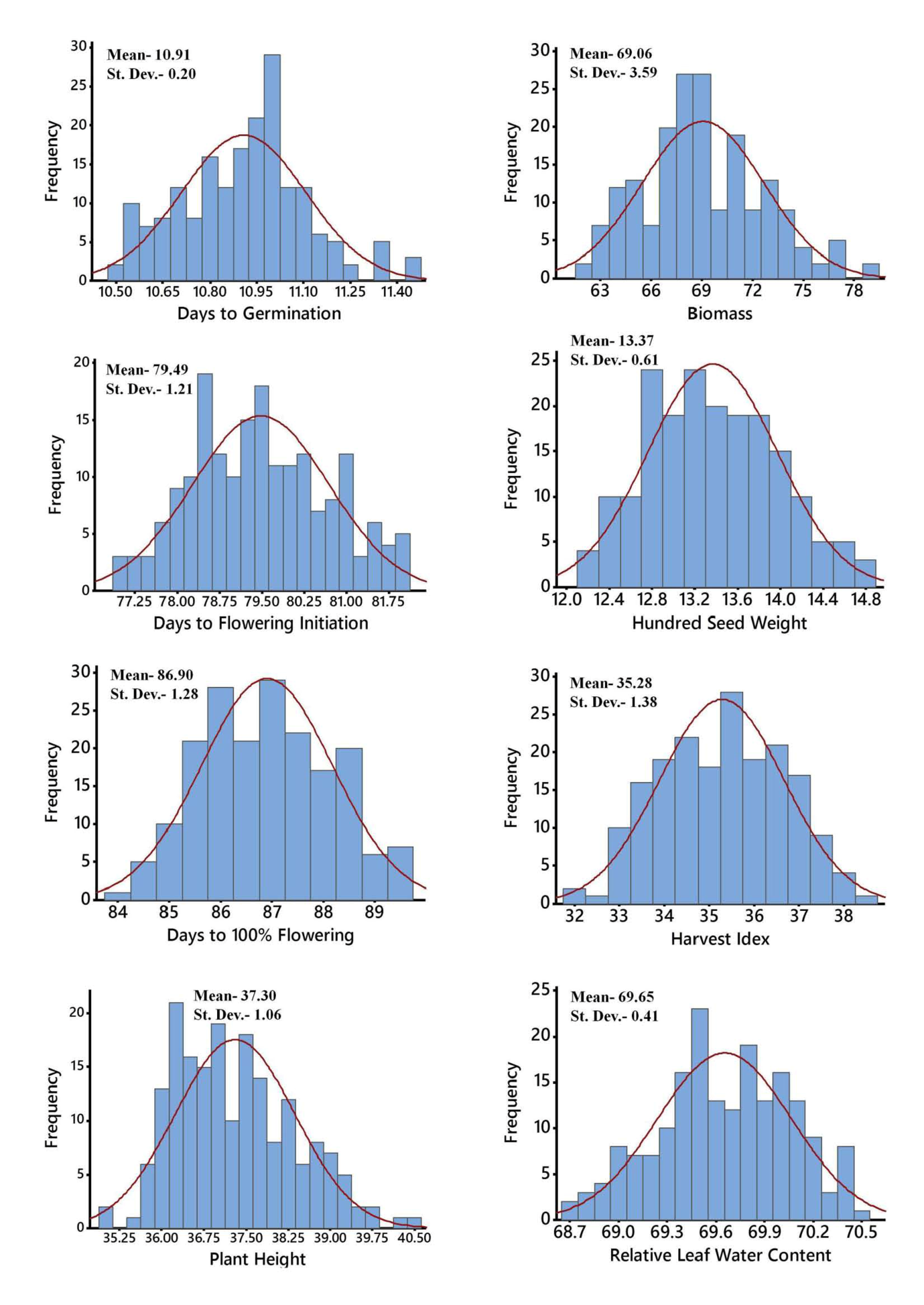

Supplement: S1 Fig — (TIF) [file pone.0254957.s001.tif]

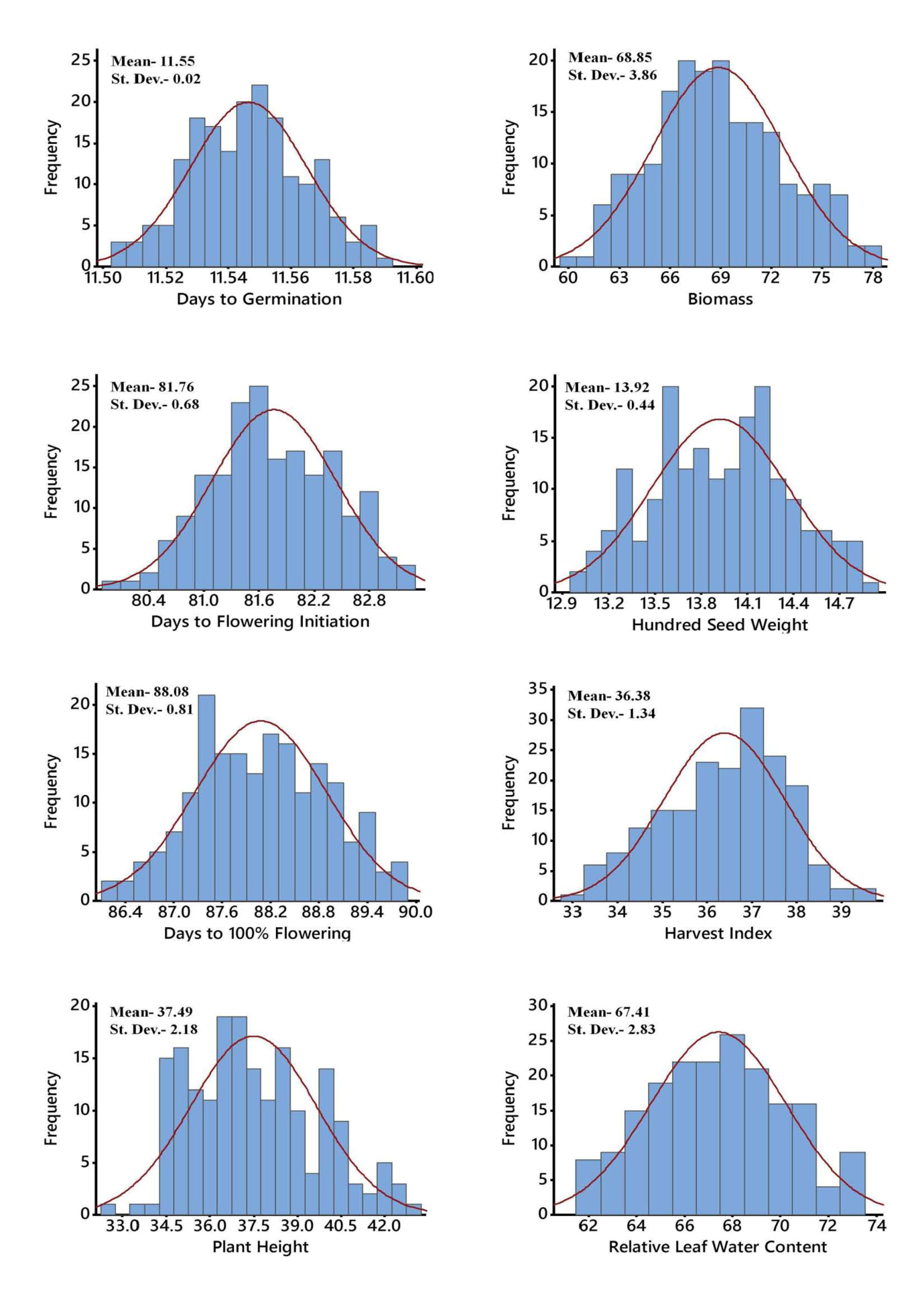

Supplement: S2 Fig — (TIF) [file pone.0254957.s002.tif]

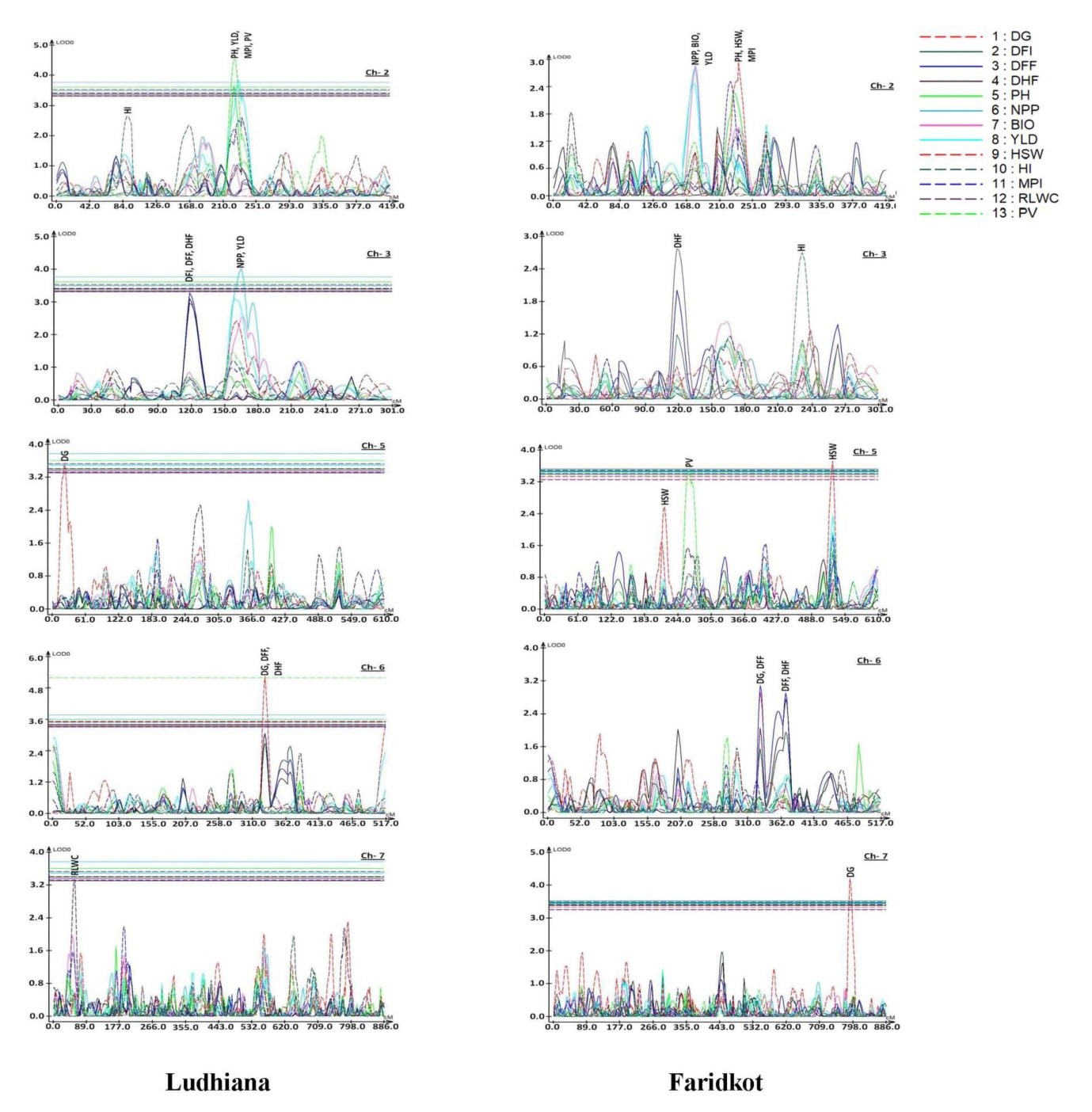

Supplement: S3 Fig — (JPG) [file pone.0254957.s003.jpg]
