# Supplementary material for: Identification of stable heat tolerance QTLs using inter-specific recombinant inbred line population derived from GPF 2 and ILWC 292
Source: PLoS One. 2021 Aug 9;16(8):e0254957. doi: 10.1371/journal.pone.0254957 (PMC8352073; doi:10.1371/journal.pone.0254957)
Supplement: S1 Table — (DOCX) [file pone.0254957.s004.docx]

**S1 Table. Best linear unbiased prediction value (BLUPs) for RIL population of pooled phenotypic data between timely-sown and late-sown conditions at Ludhiana**

| **RILs** | **Days to**  **germination** | **Days to**  **flower**  **initiation** | **Days to 50%**  **flowering** | **Days to**  **100%**  **flowering** | **Plant**  **height** | **Number**  **of**  **pods**  **per**  **plant** | **Biomass** | **Yield** | **100**  **seed**  **weight** | **Harvest index** | **Membrane**  **permeability**  **index** | **Relative**  **leaf**  **water**  **content** | **Pollen viability** |
| --- | --- | --- | --- | --- | --- | --- | --- | --- | --- | --- | --- | --- | --- |
| **1** | 10.89 | 79.04 | 82.47 | 86.12 | 36.41 | 38.49 | 68.40 | 24.96 | 13.43 | 34.45 | 43.65 | 69.45 | 77.72 |
| **2** | 11.02 | 79.13 | 82.75 | 86.31 | 38.42 | 46.08 | 75.81 | 30.28 | 14.46 | 37.14 | 40.36 | 69.94 | 82.47 |
| **3** | 11.16 | 80.79 | 84.45 | 87.70 | 38.94 | 42.00 | 74.61 | 29.82 | 13.65 | 37.30 | 40.39 | 70.05 | 83.72 |
| **4** | 10.93 | 79.59 | 83.32 | 86.68 | 38.12 | 41.94 | 73.34 | 29.03 | 14.03 | 37.16 | 40.61 | 70.14 | 83.62 |
| **5** | 10.89 | 78.58 | 82.28 | 85.57 | 38.96 | 39.84 | 73.62 | 28.66 | 14.17 | 36.71 | 40.09 | 70.32 | 82.83 |
| **6** | 10.93 | 77.93 | 81.72 | 85.19 | 37.61 | 36.32 | 67.80 | 25.96 | 13.70 | 36.99 | 42.55 | 69.79 | 78.35 |
| **7** | 10.80 | 77.75 | 81.25 | 84.64 | 37.63 | 43.09 | 73.52 | 29.36 | 13.84 | 37.34 | 41.06 | 70.19 | 81.42 |
| **8** | 11.34 | 79.32 | 83.32 | 86.77 | 38.36 | 35.81 | 68.81 | 24.27 | 13.23 | 34.44 | 43.91 | 70.21 | 79.12 |
| **9** | 11.20 | 78.95 | 82.47 | 85.94 | 35.97 | 35.06 | 64.79 | 23.44 | 12.94 | 34.76 | 43.60 | 69.40 | 77.92 |
| **10** | 11.07 | 79.13 | 82.75 | 86.22 | 36.76 | 36.54 | 67.28 | 25.17 | 12.92 | 35.12 | 44.54 | 69.58 | 77.73 |
| **11** | 10.71 | 77.29 | 81.72 | 85.29 | 36.13 | 34.06 | 64.28 | 21.92 | 12.83 | 33.49 | 45.52 | 69.24 | 76.66 |
| **12** | 10.62 | 77.56 | 81.25 | 84.82 | 38.84 | 40.75 | 74.42 | 28.47 | 13.90 | 36.20 | 41.02 | 70.19 | 82.51 |
| **13** | 11.43 | 79.59 | 83.41 | 86.68 | 37.12 | 36.96 | 67.94 | 23.98 | 13.21 | 34.54 | 43.36 | 69.51 | 76.48 |
| **14** | 10.71 | 78.49 | 82.28 | 85.57 | 39.71 | 42.08 | 76.71 | 30.35 | 14.40 | 36.82 | 40.22 | 70.11 | 83.47 |
| **15** | 10.80 | 79.50 | 83.32 | 87.24 | 37.45 | 39.42 | 70.79 | 26.80 | 13.80 | 36.03 | 42.07 | 69.61 | 78.57 |
| **16** | 10.71 | 78.76 | 82.47 | 85.10 | 35.01 | 32.65 | 61.82 | 20.78 | 12.38 | 33.23 | 45.46 | 69.30 | 76.01 |
| **17** | 10.93 | 78.58 | 82.28 | 85.75 | 36.98 | 35.41 | 66.93 | 23.59 | 12.94 | 34.50 | 44.46 | 69.88 | 76.94 |
| **18** | 10.84 | 79.59 | 83.22 | 86.77 | 37.42 | 38.31 | 69.63 | 25.49 | 13.33 | 35.43 | 43.29 | 69.71 | 79.09 |
| **19** | 10.93 | 80.06 | 83.98 | 87.42 | 36.91 | 37.83 | 68.78 | 24.39 | 12.99 | 34.66 | 44.07 | 69.78 | 77.42 |
| **20** | 10.80 | 78.58 | 82.19 | 86.59 | 37.46 | 37.91 | 69.28 | 25.22 | 13.41 | 35.20 | 44.01 | 69.77 | 78.37 |
| **21** | 10.75 | 77.29 | 80.88 | 84.36 | 35.60 | 34.92 | 64.14 | 23.04 | 12.84 | 34.67 | 45.99 | 69.52 | 77.02 |
| **22** | 11.07 | 80.15 | 83.69 | 87.61 | 36.09 | 35.19 | 65.24 | 22.90 | 12.76 | 34.00 | 45.50 | 69.11 | 76.41 |
| **23** | 11.16 | 80.06 | 83.98 | 87.61 | 36.72 | 35.75 | 66.66 | 23.24 | 12.50 | 33.78 | 44.62 | 69.21 | 76.71 |
| **24** | 10.80 | 77.10 | 80.78 | 84.64 | 36.13 | 32.30 | 63.33 | 20.26 | 12.18 | 31.85 | 46.35 | 69.29 | 75.80 |
| **25** | 11.02 | 78.58 | 82.19 | 85.57 | 36.05 | 34.46 | 64.40 | 20.90 | 12.35 | 32.17 | 45.80 | 69.10 | 75.71 |
| **26** | 10.89 | 78.49 | 82.28 | 85.75 | 38.97 | 39.64 | 73.24 | 29.57 | 14.27 | 37.57 | 40.84 | 70.42 | 83.11 |
| **27** | 10.62 | 77.01 | 80.69 | 84.08 | 36.74 | 35.97 | 66.87 | 23.23 | 12.54 | 33.68 | 43.85 | 69.66 | 76.39 |
| **28** | 10.84 | 78.21 | 81.91 | 85.57 | 35.86 | 33.37 | 63.73 | 21.38 | 12.65 | 33.17 | 44.93 | 69.17 | 76.18 |
| **29** | 10.75 | 78.40 | 82.28 | 86.03 | 35.98 | 35.75 | 65.59 | 23.54 | 12.80 | 34.40 | 44.40 | 69.48 | 77.50 |
| **30** | 10.93 | 79.04 | 82.66 | 86.31 | 35.93 | 32.26 | 62.76 | 21.06 | 12.52 | 33.10 | 46.41 | 69.49 | 75.19 |
| **31** | 10.53 | 77.75 | 81.72 | 85.10 | 36.46 | 33.57 | 64.95 | 22.96 | 12.80 | 34.11 | 45.34 | 69.07 | 76.45 |
| **32** | 10.57 | 78.40 | 82.19 | 85.66 | 37.45 | 37.38 | 69.38 | 24.09 | 12.98 | 34.08 | 44.01 | 69.47 | 77.34 |
| **33** | 10.71 | 77.93 | 81.63 | 84.91 | 35.93 | 34.92 | 64.80 | 24.00 | 12.53 | 35.29 | 44.90 | 69.35 | 75.91 |
| **34** | 10.75 | 78.67 | 82.47 | 86.03 | 37.31 | 38.11 | 69.24 | 24.73 | 13.06 | 34.79 | 42.04 | 69.78 | 78.91 |
| **35** | 10.66 | 79.41 | 83.13 | 86.68 | 36.52 | 34.20 | 65.16 | 23.33 | 12.81 | 34.42 | 45.36 | 68.79 | 77.58 |
| **36** | 10.62 | 78.12 | 81.81 | 85.10 | 37.39 | 34.80 | 66.59 | 23.35 | 12.82 | 33.97 | 44.63 | 69.35 | 77.56 |
| **37** | 10.84 | 79.78 | 83.51 | 86.87 | 36.96 | 37.34 | 68.78 | 26.20 | 12.96 | 35.37 | 44.26 | 69.67 | 78.62 |
| **38** | 11.11 | 80.06 | 83.88 | 87.52 | 35.96 | 36.13 | 65.77 | 22.85 | 13.22 | 33.63 | 44.69 | 68.82 | 77.62 |
| **39** | 11.02 | 80.89 | 84.92 | 88.54 | 36.14 | 34.98 | 64.85 | 22.24 | 13.11 | 33.47 | 44.60 | 69.33 | 75.44 |
| **40** | 11.11 | 81.53 | 85.29 | 88.73 | 37.09 | 36.76 | 67.72 | 22.98 | 13.37 | 33.56 | 44.08 | 69.36 | 77.15 |
| **41** | 11.07 | 80.52 | 84.35 | 88.17 | 36.41 | 38.25 | 68.32 | 24.46 | 13.14 | 34.06 | 43.91 | 69.32 | 77.87 |
| **42** | 10.66 | 78.21 | 82.19 | 85.47 | 36.89 | 34.62 | 65.89 | 24.65 | 13.16 | 35.39 | 45.14 | 69.55 | 76.91 |
| **43** | 10.93 | 78.86 | 82.66 | 86.22 | 38.45 | 38.74 | 71.73 | 27.40 | 14.04 | 36.38 | 41.41 | 69.90 | 80.76 |
| **44** | 10.80 | 79.41 | 83.32 | 86.96 | 37.74 | 39.28 | 71.04 | 26.62 | 13.72 | 35.84 | 42.77 | 69.84 | 79.93 |
| **45** | 10.84 | 79.41 | 83.32 | 86.87 | 37.63 | 35.63 | 67.82 | 24.51 | 13.18 | 35.17 | 42.97 | 70.04 | 80.27 |
| **46** | 10.57 | 77.93 | 81.81 | 85.29 | 37.90 | 41.62 | 72.81 | 28.25 | 14.39 | 36.69 | 41.80 | 70.30 | 83.10 |
| **47** | 10.93 | 79.13 | 83.13 | 86.87 | 37.81 | 41.18 | 72.61 | 27.10 | 13.95 | 35.70 | 41.97 | 70.11 | 81.99 |
| **48** | 10.57 | 78.49 | 82.19 | 85.94 | 38.71 | 41.38 | 73.89 | 28.66 | 14.29 | 36.56 | 41.22 | 70.04 | 83.19 |
| **49** | 11.11 | 79.96 | 83.88 | 87.61 | 38.20 | 35.99 | 68.92 | 24.04 | 13.11 | 34.09 | 44.25 | 69.96 | 78.71 |
| **50** | 10.84 | 78.58 | 82.28 | 85.75 | 36.54 | 36.78 | 67.58 | 24.56 | 13.22 | 35.29 | 43.52 | 69.53 | 77.84 |
| **51** | 10.80 | 78.03 | 81.81 | 85.38 | 38.56 | 44.56 | 76.83 | 30.10 | 14.15 | 36.58 | 40.19 | 70.20 | 84.12 |
| **52** | 10.75 | 78.21 | 82.00 | 85.57 | 37.88 | 37.22 | 69.69 | 24.80 | 13.58 | 34.55 | 43.62 | 70.08 | 79.77 |
| **53** | 10.66 | 78.76 | 82.19 | 85.75 | 38.82 | 44.34 | 76.93 | 31.36 | 14.54 | 37.48 | 39.82 | 70.13 | 84.19 |
| **54** | 10.71 | 77.38 | 81.35 | 85.10 | 40.16 | 42.04 | 77.23 | 31.23 | 14.62 | 37.29 | 40.26 | 69.73 | 84.37 |
| **55** | 10.84 | 77.93 | 81.91 | 85.29 | 38.63 | 40.57 | 73.27 | 29.86 | 14.21 | 37.95 | 41.14 | 70.20 | 83.84 |
| **56** | 10.98 | 79.04 | 82.94 | 86.68 | 39.53 | 41.50 | 75.26 | 29.91 | 14.32 | 36.99 | 41.88 | 69.86 | 83.99 |
| **57** | 10.80 | 77.93 | 81.81 | 85.38 | 39.04 | 41.40 | 74.45 | 29.54 | 14.05 | 37.05 | 41.37 | 70.19 | 83.99 |
| **58** | 10.98 | 79.13 | 83.13 | 86.87 | 39.47 | 43.09 | 76.39 | 31.63 | 14.75 | 38.04 | 39.68 | 70.40 | 84.03 |
| **59** | 10.89 | 79.13 | 83.22 | 86.77 | 38.21 | 38.95 | 71.51 | 25.73 | 13.36 | 34.83 | 43.03 | 69.51 | 80.09 |
| **60** | 11.16 | 80.06 | 83.79 | 87.15 | 39.73 | 38.99 | 73.16 | 27.98 | 13.83 | 36.29 | 41.42 | 69.68 | 81.80 |
| **61** | 11.20 | 79.50 | 83.41 | 87.33 | 38.94 | 40.49 | 73.93 | 28.89 | 14.07 | 36.80 | 40.59 | 70.03 | 82.88 |
| **62** | 11.34 | 80.06 | 83.79 | 87.42 | 38.14 | 38.73 | 71.22 | 27.56 | 13.82 | 36.78 | 42.51 | 69.97 | 79.65 |
| **63** | 11.07 | 78.49 | 82.38 | 86.12 | 36.27 | 33.29 | 63.70 | 22.24 | 12.49 | 34.13 | 44.54 | 69.10 | 76.15 |
| **64** | 11.16 | 79.50 | 83.32 | 86.96 | 36.01 | 35.18 | 64.64 | 22.41 | 12.35 | 33.91 | 44.36 | 69.35 | 76.09 |
| **65** | 10.93 | 79.32 | 83.32 | 87.05 | 37.75 | 41.62 | 73.00 | 28.44 | 13.91 | 36.47 | 41.19 | 69.90 | 81.51 |
| **66** | 11.34 | 79.32 | 82.75 | 86.31 | 38.10 | 36.15 | 68.80 | 24.74 | 13.37 | 34.94 | 42.81 | 70.04 | 78.93 |
| **67** | 11.02 | 78.76 | 82.38 | 86.03 | 38.01 | 36.37 | 69.35 | 25.93 | 13.72 | 35.95 | 43.15 | 69.47 | 78.90 |
| **68** | 10.89 | 77.66 | 81.35 | 85.19 | 39.17 | 40.17 | 73.85 | 29.43 | 14.29 | 37.06 | 40.81 | 69.96 | 82.16 |
| **69** | 10.71 | 77.20 | 80.97 | 84.64 | 40.44 | 43.35 | 78.83 | 31.18 | 14.57 | 36.63 | 40.22 | 70.41 | 84.21 |
| **70** | 10.66 | 78.30 | 82.19 | 85.94 | 39.17 | 46.46 | 78.84 | 32.88 | 14.75 | 37.78 | 39.29 | 70.41 | 84.06 |
| **71** | 11.20 | 79.13 | 82.85 | 86.68 | 39.17 | 40.77 | 74.26 | 28.75 | 14.03 | 36.48 | 41.26 | 69.75 | 83.31 |
| **72** | 10.62 | 77.38 | 81.25 | 84.91 | 37.82 | 40.18 | 71.58 | 27.93 | 13.89 | 36.76 | 41.62 | 70.04 | 78.77 |
| **73** | 10.57 | 78.21 | 81.81 | 85.47 | 38.99 | 38.85 | 72.65 | 28.78 | 14.15 | 37.26 | 41.29 | 70.10 | 83.03 |
| **74** | 10.71 | 78.12 | 81.81 | 85.47 | 38.46 | 40.21 | 72.24 | 27.92 | 13.88 | 36.74 | 41.93 | 69.90 | 83.63 |
| **75** | 10.57 | 77.84 | 81.81 | 85.29 | 37.75 | 39.66 | 71.31 | 26.52 | 13.66 | 35.71 | 42.56 | 70.41 | 81.74 |
| **76** | 10.48 | 76.92 | 80.59 | 84.45 | 39.25 | 44.12 | 77.14 | 31.75 | 14.75 | 37.66 | 40.28 | 70.48 | 84.37 |
| **77** | 10.66 | 78.49 | 82.19 | 85.94 | 38.21 | 36.17 | 69.30 | 26.29 | 13.28 | 36.48 | 42.35 | 70.41 | 80.52 |
| **78** | 10.80 | 79.41 | 83.04 | 86.68 | 38.65 | 40.49 | 73.37 | 28.32 | 14.22 | 36.47 | 41.97 | 70.41 | 82.92 |
| **79** | 10.89 | 79.78 | 83.69 | 87.15 | 37.14 | 37.34 | 68.60 | 24.16 | 13.12 | 34.43 | 42.91 | 70.03 | 78.73 |
| **80** | 10.62 | 78.67 | 82.57 | 86.03 | 38.43 | 35.57 | 69.15 | 25.99 | 13.12 | 36.19 | 43.38 | 69.98 | 79.58 |
| **81** | 10.89 | 77.75 | 81.63 | 85.38 | 39.20 | 43.80 | 74.28 | 30.96 | 14.35 | 38.59 | 40.59 | 70.21 | 84.27 |
| **82** | 11.07 | 80.89 | 84.82 | 88.35 | 37.49 | 40.11 | 70.75 | 27.49 | 13.71 | 36.79 | 42.10 | 69.79 | 81.01 |
| **83** | 11.02 | 81.53 | 85.57 | 89.19 | 36.99 | 33.83 | 66.71 | 21.85 | 12.53 | 32.48 | 44.17 | 69.37 | 76.79 |
| **84** | 10.89 | 80.33 | 84.26 | 87.98 | 37.54 | 38.49 | 69.87 | 26.71 | 13.65 | 36.57 | 42.38 | 69.48 | 78.61 |
| **85** | 11.11 | 81.62 | 85.57 | 89.19 | 37.04 | 39.16 | 69.80 | 26.97 | 13.87 | 36.65 | 42.90 | 69.78 | 79.65 |
| **86** | 10.80 | 79.87 | 83.79 | 87.61 | 38.34 | 37.64 | 69.69 | 26.91 | 13.88 | 36.90 | 41.30 | 69.92 | 82.12 |
| **87** | 11.11 | 82.09 | 86.04 | 89.66 | 38.52 | 35.27 | 68.20 | 23.92 | 13.12 | 34.26 | 43.35 | 70.06 | 80.14 |
| **88** | 10.75 | 80.24 | 83.98 | 87.61 | 37.50 | 39.08 | 70.72 | 27.28 | 13.51 | 36.64 | 41.92 | 69.61 | 81.56 |
| **89** | 10.93 | 79.78 | 83.69 | 87.33 | 36.68 | 37.12 | 67.76 | 24.49 | 13.29 | 34.99 | 43.35 | 69.55 | 78.10 |
| **90** | 10.98 | 79.59 | 83.32 | 86.87 | 36.65 | 35.87 | 67.23 | 24.68 | 12.93 | 34.67 | 43.04 | 68.90 | 78.10 |
| **91** | 10.71 | 79.50 | 83.41 | 86.96 | 36.16 | 34.05 | 64.79 | 23.09 | 12.85 | 34.85 | 44.26 | 68.74 | 76.30 |
| **92** | 10.57 | 78.76 | 82.57 | 86.12 | 37.95 | 39.68 | 73.08 | 27.01 | 13.44 | 34.41 | 42.12 | 69.00 | 78.61 |
| **93** | 10.57 | 78.30 | 82.28 | 85.94 | 37.41 | 41.32 | 72.46 | 27.59 | 13.69 | 35.82 | 42.36 | 69.44 | 79.69 |
| **94** | 10.75 | 80.61 | 84.54 | 88.45 | 38.30 | 41.05 | 70.97 | 26.85 | 13.77 | 36.41 | 42.63 | 70.29 | 81.31 |
| **95** | 10.89 | 81.07 | 85.01 | 88.63 | 37.87 | 35.49 | 67.29 | 24.94 | 13.34 | 36.12 | 43.43 | 70.40 | 79.08 |
| **96** | 10.98 | 80.52 | 84.35 | 87.89 | 36.17 | 35.28 | 65.19 | 22.75 | 13.03 | 34.25 | 44.10 | 69.67 | 76.27 |
| **97** | 10.48 | 78.58 | 82.38 | 85.94 | 37.34 | 37.08 | 68.88 | 24.14 | 13.27 | 34.12 | 43.57 | 69.96 | 77.28 |
| **98** | 10.57 | 80.98 | 85.01 | 88.73 | 36.72 | 36.21 | 67.46 | 23.65 | 12.90 | 34.23 | 44.52 | 69.79 | 77.44 |
| **99** | 10.93 | 80.06 | 83.98 | 87.70 | 36.85 | 37.77 | 68.72 | 25.24 | 13.35 | 35.33 | 43.26 | 69.55 | 78.91 |
| **100** | 10.62 | 80.89 | 84.63 | 88.63 | 36.21 | 35.73 | 65.44 | 23.78 | 13.04 | 35.36 | 43.55 | 68.92 | 76.89 |
| **101** | 10.89 | 80.61 | 84.45 | 88.17 | 36.57 | 35.59 | 65.89 | 22.32 | 12.46 | 33.35 | 44.11 | 69.16 | 76.79 |
| **102** | 11.02 | 80.89 | 83.88 | 87.42 | 36.85 | 35.91 | 66.67 | 23.49 | 12.88 | 33.96 | 44.37 | 68.81 | 76.94 |
| **103** | 10.93 | 80.24 | 84.26 | 88.17 | 37.12 | 37.89 | 70.59 | 26.15 | 13.64 | 34.66 | 42.36 | 69.37 | 78.52 |
| **104** | 11.20 | 80.79 | 84.82 | 88.35 | 36.75 | 37.97 | 68.33 | 25.57 | 13.17 | 35.07 | 42.80 | 69.47 | 78.60 |
| **105** | 11.11 | 81.35 | 85.01 | 88.73 | 36.89 | 35.51 | 67.25 | 24.57 | 13.10 | 35.29 | 42.97 | 69.95 | 78.47 |
| **106** | 10.98 | 81.81 | 85.76 | 89.38 | 35.84 | 35.16 | 64.49 | 21.79 | 12.65 | 33.42 | 45.29 | 69.63 | 76.77 |
| **107** | 10.89 | 80.42 | 84.16 | 87.70 | 35.89 | 35.00 | 63.04 | 22.11 | 12.88 | 35.37 | 44.37 | 68.97 | 76.59 |
| **108** | 10.93 | 80.33 | 84.16 | 87.80 | 36.54 | 37.91 | 67.73 | 24.19 | 12.86 | 34.31 | 44.26 | 69.27 | 77.83 |
| **109** | 11.02 | 81.44 | 85.10 | 88.63 | 37.31 | 38.87 | 69.92 | 25.99 | 13.45 | 35.41 | 43.39 | 69.50 | 77.37 |
| **110** | 10.89 | 78.49 | 82.38 | 85.94 | 37.15 | 36.58 | 68.38 | 24.82 | 13.63 | 35.05 | 44.02 | 69.87 | 77.21 |
| **111** | 10.98 | 79.59 | 83.41 | 86.96 | 36.36 | 36.66 | 66.33 | 24.23 | 12.82 | 34.83 | 44.17 | 69.15 | 77.20 |
| **112** | 10.66 | 77.66 | 81.35 | 85.01 | 37.04 | 37.30 | 68.32 | 24.40 | 12.77 | 34.51 | 42.86 | 69.82 | 76.91 |
| **113** | 10.57 | 80.06 | 83.69 | 87.24 | 37.66 | 36.09 | 68.81 | 23.45 | 13.04 | 33.57 | 44.05 | 69.35 | 78.22 |
| **114** | 10.93 | 78.30 | 82.19 | 85.84 | 37.61 | 37.85 | 69.48 | 24.62 | 13.32 | 34.55 | 43.18 | 69.53 | 78.55 |
| **115** | 11.02 | 81.07 | 84.82 | 88.63 | 37.01 | 39.48 | 69.89 | 26.38 | 13.63 | 36.05 | 42.40 | 69.48 | 80.18 |
| **116** | 11.07 | 79.23 | 83.04 | 86.77 | 37.54 | 41.44 | 72.15 | 27.68 | 13.90 | 36.11 | 41.99 | 70.09 | 79.78 |
| **117** | 10.84 | 78.30 | 82.19 | 86.03 | 38.31 | 38.71 | 71.30 | 27.91 | 13.91 | 37.06 | 41.39 | 70.13 | 81.74 |
| **118** | 10.89 | 78.86 | 82.75 | 86.31 | 36.23 | 38.71 | 67.60 | 25.90 | 13.22 | 36.63 | 43.27 | 69.35 | 78.65 |
| **119** | 10.93 | 78.12 | 81.63 | 85.29 | 37.08 | 39.08 | 69.34 | 26.39 | 13.71 | 36.05 | 41.87 | 69.64 | 79.23 |
| **120** | 10.89 | 79.87 | 83.41 | 87.05 | 37.95 | 37.06 | 68.85 | 25.29 | 13.47 | 35.64 | 42.61 | 69.55 | 79.53 |
| **121** | 10.93 | 79.04 | 83.04 | 86.87 | 36.77 | 36.98 | 67.84 | 23.02 | 12.87 | 33.44 | 44.05 | 69.46 | 77.27 |
| **122** | 11.11 | 80.15 | 83.69 | 87.33 | 36.24 | 35.75 | 64.43 | 23.00 | 13.07 | 35.49 | 43.68 | 69.58 | 76.71 |
| **123** | 10.84 | 79.23 | 83.13 | 86.96 | 37.68 | 38.95 | 70.95 | 26.71 | 14.01 | 35.69 | 43.08 | 69.84 | 79.01 |
| **124** | 11.43 | 81.72 | 85.67 | 89.28 | 36.63 | 43.53 | 72.06 | 29.37 | 13.49 | 36.72 | 42.01 | 68.98 | 78.58 |
| **125** | 10.98 | 78.95 | 82.66 | 86.31 | 36.28 | 32.99 | 63.61 | 21.51 | 12.78 | 33.24 | 45.45 | 68.99 | 76.22 |
| **126** | 11.25 | 79.50 | 83.32 | 87.05 | 37.03 | 36.61 | 68.09 | 23.15 | 12.75 | 33.48 | 44.52 | 69.03 | 76.86 |
| **127** | 10.93 | 78.40 | 82.10 | 85.66 | 37.37 | 41.76 | 71.98 | 28.66 | 13.98 | 37.14 | 41.17 | 70.08 | 81.94 |
| **128** | 11.34 | 78.76 | 82.38 | 86.31 | 36.57 | 38.41 | 67.84 | 26.14 | 13.43 | 35.96 | 42.78 | 69.45 | 78.57 |
| **129** | 11.07 | 79.13 | 82.57 | 86.31 | 36.16 | 38.33 | 67.47 | 26.14 | 13.42 | 36.25 | 42.79 | 69.44 | 78.45 |
| **130** | 11.11 | 78.95 | 82.85 | 86.50 | 38.16 | 40.91 | 75.22 | 28.92 | 13.79 | 35.12 | 41.21 | 70.14 | 79.45 |
| **131** | 10.80 | 79.78 | 83.41 | 87.05 | 38.96 | 41.74 | 73.35 | 30.19 | 14.62 | 38.23 | 40.75 | 70.24 | 83.76 |
| **132** | 11.11 | 80.52 | 84.07 | 88.08 | 38.63 | 38.39 | 70.61 | 27.35 | 13.77 | 36.89 | 42.24 | 70.13 | 80.70 |
| **133** | 10.98 | 79.78 | 83.69 | 87.15 | 36.53 | 35.12 | 64.35 | 22.92 | 13.26 | 35.45 | 44.60 | 69.61 | 76.56 |
| **134** | 10.98 | 80.70 | 84.63 | 87.98 | 36.85 | 37.46 | 68.45 | 25.48 | 13.58 | 35.67 | 42.96 | 69.83 | 79.01 |
| **135** | 10.71 | 78.40 | 82.00 | 85.66 | 37.75 | 38.86 | 70.82 | 26.54 | 13.78 | 35.67 | 42.73 | 69.72 | 78.67 |
| **136** | 10.62 | 80.33 | 84.16 | 87.80 | 37.44 | 35.91 | 68.59 | 24.37 | 13.21 | 34.48 | 43.21 | 69.79 | 78.35 |
| **137** | 10.80 | 79.13 | 82.85 | 86.31 | 35.82 | 33.05 | 63.26 | 20.98 | 12.27 | 32.77 | 45.31 | 68.95 | 76.07 |
| **138** | 10.80 | 79.78 | 83.60 | 87.33 | 36.22 | 34.64 | 64.59 | 22.38 | 12.53 | 34.00 | 44.69 | 68.97 | 76.08 |
| **139** | 10.75 | 78.76 | 82.66 | 86.31 | 35.84 | 33.71 | 63.49 | 21.42 | 12.40 | 33.26 | 46.01 | 69.12 | 75.37 |
| **140** | 10.71 | 78.76 | 82.57 | 85.94 | 38.69 | 37.81 | 70.68 | 25.79 | 13.55 | 35.31 | 42.59 | 69.98 | 80.98 |
| **141** | 10.66 | 78.86 | 82.66 | 86.40 | 37.59 | 34.70 | 67.53 | 23.15 | 12.86 | 33.72 | 44.63 | 69.26 | 77.80 |
| **142** | 10.71 | 78.40 | 82.10 | 85.66 | 37.50 | 41.23 | 71.88 | 28.57 | 14.05 | 37.15 | 41.77 | 69.80 | 80.97 |
| **143** | 10.84 | 78.40 | 82.19 | 85.75 | 37.87 | 40.16 | 70.78 | 27.94 | 14.06 | 37.45 | 42.19 | 69.89 | 81.67 |
| **144** | 10.89 | 79.13 | 83.22 | 86.59 | 37.47 | 38.15 | 69.49 | 25.09 | 13.56 | 34.98 | 42.93 | 69.84 | 77.56 |
| **145** | 10.93 | 78.40 | 82.10 | 85.94 | 37.02 | 36.80 | 67.86 | 23.75 | 12.94 | 34.30 | 44.34 | 69.47 | 76.93 |
| **146** | 11.02 | 80.15 | 83.88 | 87.61 | 37.59 | 36.82 | 68.75 | 25.43 | 13.20 | 35.70 | 43.60 | 69.75 | 80.26 |
| **147** | 11.16 | 79.69 | 83.32 | 86.87 | 37.09 | 36.01 | 67.85 | 23.83 | 12.95 | 34.38 | 43.84 | 69.66 | 78.28 |
| **148** | 11.07 | 81.35 | 85.01 | 88.73 | 36.74 | 38.25 | 67.74 | 25.52 | 13.47 | 36.43 | 42.50 | 69.86 | 79.41 |
| **149** | 11.07 | 80.89 | 84.73 | 88.17 | 35.88 | 33.83 | 63.67 | 21.45 | 12.28 | 33.11 | 44.79 | 68.90 | 76.15 |
| **150** | 11.47 | 81.90 | 85.86 | 89.56 | 35.12 | 31.80 | 61.64 | 20.95 | 12.32 | 33.40 | 46.19 | 69.19 | 76.09 |
| **151** | 11.34 | 81.99 | 85.86 | 89.75 | 36.18 | 34.72 | 64.98 | 22.35 | 12.53 | 33.55 | 45.99 | 68.99 | 76.39 |
| **152** | 11.07 | 81.62 | 85.57 | 89.19 | 37.35 | 35.69 | 69.02 | 24.19 | 12.84 | 33.82 | 44.06 | 69.54 | 77.45 |
| **153** | 10.75 | 80.33 | 84.16 | 87.89 | 37.17 | 35.93 | 68.29 | 23.54 | 12.87 | 33.77 | 43.95 | 69.52 | 77.16 |
| **154** | 10.80 | 80.61 | 84.54 | 88.17 | 36.67 | 35.33 | 66.75 | 22.24 | 12.50 | 32.95 | 44.71 | 69.27 | 76.26 |
| **155** | 10.80 | 80.06 | 83.88 | 87.33 | 36.02 | 40.53 | 69.33 | 26.41 | 13.02 | 35.41 | 43.46 | 69.54 | 78.60 |
| **156** | 10.98 | 79.96 | 83.98 | 87.70 | 35.70 | 32.99 | 62.96 | 21.27 | 12.25 | 33.09 | 45.66 | 69.00 | 76.20 |
| **157** | 10.93 | 80.70 | 84.35 | 87.80 | 36.21 | 32.50 | 62.99 | 21.09 | 12.36 | 32.93 | 46.42 | 69.12 | 75.36 |
| **158** | 10.98 | 80.70 | 84.35 | 87.98 | 36.15 | 34.28 | 64.57 | 22.89 | 12.82 | 34.31 | 44.40 | 69.39 | 76.72 |
| **159** | 10.93 | 81.72 | 85.57 | 89.19 | 36.43 | 37.18 | 67.41 | 25.76 | 13.21 | 35.52 | 43.80 | 69.44 | 78.53 |
| **160** | 10.98 | 81.44 | 85.20 | 88.73 | 36.60 | 37.08 | 67.21 | 24.18 | 12.69 | 34.76 | 42.95 | 69.85 | 78.38 |
| **161** | 10.98 | 80.33 | 84.16 | 87.80 | 36.58 | 39.86 | 71.21 | 27.12 | 13.63 | 35.37 | 42.64 | 70.04 | 79.14 |
| **162** | 11.02 | 81.25 | 85.10 | 88.91 | 35.92 | 38.53 | 67.82 | 25.68 | 13.44 | 35.27 | 44.47 | 69.39 | 78.72 |
| **163** | 10.84 | 80.70 | 84.82 | 88.26 | 36.30 | 36.53 | 66.68 | 25.26 | 12.91 | 35.56 | 44.72 | 69.13 | 78.51 |
| **164** | 11.20 | 80.06 | 83.88 | 87.61 | 36.36 | 39.22 | 68.22 | 25.95 | 13.28 | 35.54 | 42.91 | 69.38 | 78.87 |
| **165** | 10.89 | 79.87 | 83.88 | 87.80 | 35.80 | 35.33 | 64.46 | 22.76 | 12.70 | 34.23 | 44.94 | 69.17 | 76.66 |
| **166** | 11.07 | 81.99 | 85.76 | 89.28 | 36.15 | 33.87 | 64.45 | 21.51 | 12.51 | 32.79 | 45.43 | 68.69 | 76.58 |
| **167** | 10.98 | 80.89 | 84.54 | 88.35 | 36.24 | 35.31 | 65.60 | 24.05 | 12.92 | 34.86 | 43.96 | 69.31 | 78.05 |
| **168** | 11.02 | 80.15 | 84.16 | 87.89 | 36.51 | 39.58 | 71.01 | 27.11 | 13.96 | 35.86 | 42.40 | 69.83 | 80.02 |
| **169** | 10.98 | 81.07 | 84.92 | 88.54 | 37.04 | 39.80 | 70.87 | 26.48 | 13.65 | 35.36 | 42.99 | 69.59 | 79.29 |
| **170** | 11.02 | 79.50 | 83.51 | 87.42 | 36.05 | 37.28 | 66.95 | 26.06 | 13.23 | 36.06 | 43.31 | 69.51 | 78.56 |
| **171** | 11.02 | 79.41 | 83.41 | 87.05 | 37.13 | 37.99 | 69.24 | 26.00 | 13.64 | 35.93 | 42.87 | 69.81 | 78.57 |
| **172** | 10.80 | 78.30 | 82.10 | 85.84 | 38.22 | 36.96 | 69.60 | 25.70 | 13.71 | 35.70 | 43.16 | 69.70 | 79.69 |
| **173** | 10.84 | 79.04 | 82.66 | 86.22 | 37.85 | 39.06 | 70.98 | 26.92 | 13.66 | 36.12 | 41.90 | 69.56 | 80.59 |
| **174** | 10.71 | 79.04 | 82.75 | 86.12 | 37.55 | 34.10 | 67.00 | 22.57 | 12.94 | 33.34 | 43.75 | 69.43 | 76.98 |
| **175** | 11.11 | 81.07 | 84.92 | 88.54 | 36.58 | 37.50 | 67.66 | 23.50 | 12.72 | 34.01 | 44.63 | 69.34 | 76.36 |
| **176** | 10.80 | 78.21 | 81.91 | 85.66 | 37.03 | 33.79 | 65.65 | 22.30 | 12.81 | 33.55 | 44.36 | 69.62 | 76.61 |
| **177** | 10.84 | 80.98 | 84.92 | 88.54 | 37.08 | 39.78 | 70.38 | 27.20 | 13.91 | 36.37 | 42.13 | 69.73 | 80.47 |
| **178** | 10.89 | 80.79 | 84.73 | 88.35 | 38.04 | 38.99 | 71.38 | 28.06 | 14.12 | 36.68 | 41.05 | 69.92 | 80.26 |
| **179** | 10.66 | 79.59 | 83.32 | 86.87 | 37.80 | 41.66 | 73.34 | 28.40 | 14.00 | 36.03 | 41.76 | 70.04 | 79.84 |
| **180** | 10.93 | 78.95 | 82.75 | 86.31 | 36.55 | 37.18 | 67.46 | 25.72 | 13.51 | 35.76 | 42.83 | 69.86 | 78.65 |
| **181** | 11.11 | 81.99 | 86.04 | 89.56 | 38.65 | 40.33 | 73.29 | 29.18 | 14.53 | 37.29 | 40.75 | 70.08 | 82.84 |
| **182** | 10.98 | 80.70 | 84.73 | 88.35 | 37.37 | 35.14 | 67.10 | 24.19 | 13.54 | 35.10 | 42.88 | 69.44 | 77.81 |
| **183** | 11.07 | 79.69 | 83.60 | 86.96 | 36.82 | 38.09 | 68.89 | 25.87 | 13.41 | 35.95 | 42.87 | 69.67 | 79.05 |
| **184** | 11.11 | 79.13 | 82.94 | 86.40 | 38.14 | 35.36 | 68.59 | 23.85 | 13.36 | 34.00 | 43.59 | 69.77 | 80.27 |
| **185** | 11.25 | 80.24 | 83.88 | 87.33 | 38.36 | 42.52 | 74.78 | 29.62 | 14.28 | 36.99 | 40.36 | 70.25 | 83.35 |
| **186** | 11.16 | 81.72 | 85.57 | 89.19 | 36.46 | 37.22 | 67.82 | 24.44 | 12.89 | 34.09 | 44.73 | 69.51 | 77.47 |
| **187** | 10.93 | 79.41 | 83.22 | 87.05 | 36.34 | 39.42 | 69.40 | 25.53 | 13.38 | 35.07 | 42.34 | 69.78 | 79.02 |
