# Supplementary material for: Identification of stable heat tolerance QTLs using inter-specific recombinant inbred line population derived from GPF 2 and ILWC 292
Source: PLoS One. 2021 Aug 9;16(8):e0254957. doi: 10.1371/journal.pone.0254957 (PMC8352073; doi:10.1371/journal.pone.0254957)
Supplement: S2 Table — (DOCX) [file pone.0254957.s005.docx]

**S2 Table. Best linear unbiased prediction value (BLUPs) for RIL population of pooled phenotypic data between timely-sown and late-sown conditions at Faridkot**

| **RILs** | **Days to**  **germination** | **Days to**  **flower**  **initiation** | **Days to 50%**  **flowering** | **Days to**  **100%**  **flowering** | **Plant**  **height** | **Number**  **of**  **pods**  **per**  **plant** | **Biomass** | **Yield** | **100**  **seed**  **weight** | **Harvest index** | **Membrane**  **permeability**  **index** | **Relative**  **leaf**  **water**  **content** | **Pollen viability** |
| --- | --- | --- | --- | --- | --- | --- | --- | --- | --- | --- | --- | --- | --- |
| **1** | 11.54 | 81.39 | 83.92 | 87.90 | 35.20 | 37.96 | 67.40 | 24.81 | 13.81 | 35.47 | 44.14 | 66.29 | 76.57 |
| **2** | 11.57 | 81.89 | 84.20 | 88.05 | 37.88 | 45.60 | 74.01 | 28.05 | 14.19 | 36.33 | 41.79 | 68.77 | 80.37 |
| **3** | 11.57 | 82.64 | 85.26 | 88.94 | 37.34 | 40.88 | 70.53 | 29.41 | 14.13 | 39.72 | 39.91 | 71.08 | 81.98 |
| **4** | 11.56 | 81.70 | 83.93 | 87.85 | 39.69 | 44.21 | 74.15 | 29.74 | 14.19 | 37.72 | 39.70 | 72.11 | 82.28 |
| **5** | 11.55 | 81.39 | 83.86 | 88.00 | 39.85 | 43.93 | 74.80 | 30.15 | 14.43 | 37.66 | 40.04 | 72.27 | 82.37 |
| **6** | 11.55 | 80.27 | 82.52 | 86.67 | 38.81 | 39.32 | 69.71 | 28.61 | 14.23 | 39.30 | 40.74 | 70.32 | 81.38 |
| **7** | 11.55 | 80.83 | 83.23 | 87.41 | 39.31 | 43.00 | 72.34 | 29.31 | 14.28 | 38.16 | 40.04 | 71.46 | 81.82 |
| **8** | 11.57 | 82.01 | 84.56 | 88.52 | 37.83 | 37.78 | 67.27 | 26.04 | 13.86 | 37.85 | 41.70 | 68.31 | 80.47 |
| **9** | 11.55 | 81.57 | 84.00 | 88.00 | 34.46 | 35.15 | 63.74 | 24.18 | 13.45 | 36.70 | 44.97 | 65.61 | 76.80 |
| **10** | 11.56 | 81.39 | 84.07 | 88.00 | 35.84 | 37.80 | 65.72 | 25.62 | 13.59 | 36.98 | 43.63 | 67.07 | 77.32 |
| **11** | 11.54 | 81.33 | 83.58 | 87.56 | 37.32 | 38.23 | 68.16 | 26.21 | 13.68 | 37.05 | 42.51 | 68.03 | 79.67 |
| **12** | 11.52 | 80.77 | 83.23 | 87.26 | 41.18 | 45.83 | 75.69 | 28.43 | 14.23 | 36.26 | 41.27 | 69.94 | 82.20 |
| **13** | 11.59 | 81.89 | 84.49 | 88.74 | 36.32 | 38.21 | 67.59 | 24.99 | 13.57 | 36.18 | 42.96 | 66.37 | 79.32 |
| **14** | 11.53 | 81.39 | 83.65 | 87.48 | 40.20 | 44.69 | 74.67 | 30.41 | 14.59 | 37.95 | 39.40 | 71.17 | 82.72 |
| **15** | 11.53 | 80.77 | 83.37 | 87.19 | 36.99 | 39.52 | 69.85 | 26.72 | 13.86 | 36.80 | 41.36 | 68.99 | 79.97 |
| **16** | 11.52 | 81.20 | 83.65 | 87.19 | 36.63 | 34.19 | 63.71 | 22.79 | 13.44 | 36.13 | 44.41 | 64.45 | 76.77 |
| **17** | 11.54 | 81.02 | 83.15 | 87.09 | 37.21 | 35.58 | 65.29 | 23.05 | 13.52 | 35.53 | 44.47 | 65.20 | 77.08 |
| **18** | 11.56 | 81.89 | 84.41 | 88.20 | 36.28 | 38.66 | 67.14 | 26.04 | 13.77 | 37.54 | 43.16 | 67.06 | 79.19 |
| **19** | 11.55 | 82.76 | 85.19 | 88.86 | 37.30 | 39.87 | 69.04 | 25.88 | 13.71 | 36.57 | 42.54 | 67.12 | 79.66 |
| **20** | 11.54 | 81.45 | 83.86 | 87.71 | 36.66 | 38.74 | 68.61 | 25.39 | 13.66 | 36.15 | 43.22 | 67.08 | 79.07 |
| **21** | 11.53 | 80.58 | 82.80 | 86.67 | 37.21 | 37.58 | 67.82 | 24.05 | 13.58 | 34.98 | 44.31 | 65.80 | 78.03 |
| **22** | 11.56 | 82.20 | 84.56 | 88.52 | 35.40 | 34.12 | 64.58 | 22.38 | 13.29 | 34.65 | 45.74 | 63.17 | 75.32 |
| **23** | 11.59 | 82.38 | 84.85 | 88.96 | 38.50 | 37.95 | 68.89 | 23.37 | 13.83 | 34.45 | 44.46 | 66.33 | 76.58 |
| **24** | 11.52 | 80.58 | 82.66 | 86.75 | 35.13 | 30.64 | 61.98 | 20.05 | 13.00 | 33.45 | 46.58 | 61.74 | 72.83 |
| **25** | 11.55 | 81.45 | 83.79 | 87.41 | 37.23 | 35.99 | 65.90 | 22.35 | 13.29 | 34.35 | 44.61 | 65.27 | 76.05 |
| **26** | 11.54 | 81.33 | 83.51 | 87.41 | 40.00 | 42.83 | 73.58 | 31.09 | 14.74 | 39.00 | 39.70 | 72.40 | 83.51 |
| **27** | 11.54 | 80.39 | 82.38 | 86.23 | 35.93 | 39.52 | 67.52 | 25.36 | 13.61 | 36.50 | 42.93 | 67.08 | 79.39 |
| **28** | 11.56 | 80.95 | 83.23 | 86.97 | 32.67 | 31.97 | 60.42 | 21.68 | 13.22 | 36.29 | 45.54 | 63.93 | 75.35 |
| **29** | 11.55 | 81.20 | 83.72 | 87.56 | 36.53 | 39.59 | 67.89 | 24.76 | 13.78 | 35.55 | 43.90 | 67.10 | 78.58 |
| **30** | 11.55 | 81.14 | 83.51 | 87.41 | 38.36 | 34.72 | 65.79 | 22.34 | 13.33 | 34.43 | 44.80 | 64.98 | 76.58 |
| **31** | 11.52 | 81.14 | 83.44 | 87.41 | 39.07 | 38.46 | 69.29 | 27.54 | 14.25 | 37.86 | 41.84 | 68.62 | 80.85 |
| **32** | 11.53 | 81.33 | 83.71 | 87.68 | 38.79 | 39.37 | 70.44 | 25.85 | 13.61 | 35.95 | 42.51 | 67.80 | 79.38 |
| **33** | 11.52 | 81.27 | 83.36 | 87.54 | 34.80 | 34.73 | 63.33 | 24.54 | 13.24 | 37.51 | 44.50 | 65.75 | 77.74 |
| **34** | 11.56 | 81.27 | 83.71 | 87.31 | 37.08 | 39.07 | 68.82 | 25.14 | 13.59 | 36.03 | 43.11 | 67.12 | 78.88 |
| **35** | 11.51 | 81.39 | 83.58 | 87.41 | 36.35 | 36.21 | 65.65 | 23.94 | 13.55 | 35.73 | 44.45 | 64.82 | 78.27 |
| **36** | 11.51 | 81.08 | 83.30 | 87.19 | 36.83 | 33.56 | 65.87 | 22.80 | 13.47 | 34.66 | 45.18 | 63.96 | 75.52 |
| **37** | 11.55 | 81.45 | 83.72 | 87.85 | 37.96 | 41.46 | 71.26 | 27.52 | 13.96 | 36.36 | 42.32 | 68.20 | 79.86 |
| **38** | 11.57 | 82.01 | 84.49 | 88.37 | 34.55 | 35.20 | 64.87 | 22.23 | 13.38 | 34.49 | 45.55 | 63.21 | 75.28 |
| **39** | 11.58 | 82.26 | 84.71 | 88.66 | 35.31 | 33.69 | 64.05 | 21.91 | 13.38 | 34.50 | 45.94 | 62.27 | 75.04 |
| **40** | 11.55 | 82.94 | 85.41 | 89.40 | 36.64 | 36.09 | 66.68 | 22.22 | 13.66 | 34.12 | 44.38 | 65.16 | 76.44 |
| **41** | 11.57 | 82.01 | 84.42 | 88.15 | 35.35 | 37.70 | 67.40 | 23.90 | 13.73 | 34.84 | 44.95 | 65.41 | 76.69 |
| **42** | 11.53 | 81.02 | 83.16 | 87.12 | 36.52 | 32.96 | 65.07 | 24.00 | 13.67 | 35.85 | 44.65 | 64.38 | 77.36 |
| **43** | 11.56 | 81.89 | 84.07 | 88.07 | 37.23 | 36.94 | 66.87 | 26.92 | 14.15 | 38.93 | 42.02 | 67.93 | 80.63 |
| **44** | 11.52 | 81.82 | 84.28 | 87.93 | 35.88 | 36.41 | 66.44 | 24.39 | 13.64 | 36.34 | 43.52 | 66.06 | 78.75 |
| **45** | 11.52 | 81.82 | 84.14 | 87.78 | 38.21 | 38.18 | 68.70 | 26.11 | 13.97 | 36.79 | 41.58 | 69.41 | 79.63 |
| **46** | 11.53 | 81.33 | 83.58 | 87.56 | 37.54 | 41.99 | 72.28 | 27.72 | 14.55 | 36.81 | 41.26 | 69.52 | 82.01 |
| **47** | 11.56 | 81.76 | 84.28 | 88.07 | 39.07 | 39.54 | 69.93 | 25.61 | 13.88 | 35.75 | 41.77 | 68.02 | 79.80 |
| **48** | 11.54 | 81.26 | 83.73 | 87.51 | 39.07 | 41.94 | 72.59 | 28.28 | 14.55 | 37.11 | 40.65 | 69.48 | 81.75 |
| **49** | 11.58 | 82.13 | 84.64 | 88.39 | 39.75 | 42.67 | 73.07 | 28.31 | 14.36 | 36.94 | 40.56 | 69.67 | 81.34 |
| **50** | 11.54 | 81.32 | 83.73 | 87.51 | 40.09 | 39.29 | 71.08 | 25.80 | 13.64 | 35.51 | 42.04 | 66.86 | 80.13 |
| **51** | 11.55 | 80.88 | 83.10 | 87.01 | 38.74 | 46.07 | 75.54 | 30.16 | 14.69 | 37.34 | 39.42 | 71.09 | 83.25 |
| **52** | 11.55 | 81.01 | 83.39 | 87.31 | 41.99 | 41.56 | 73.88 | 26.92 | 13.98 | 35.59 | 41.07 | 67.94 | 79.73 |
| **53** | 11.52 | 81.44 | 83.74 | 87.60 | 39.02 | 45.42 | 75.94 | 31.06 | 14.72 | 37.81 | 39.21 | 71.38 | 83.01 |
| **54** | 11.53 | 80.57 | 82.75 | 86.42 | 41.84 | 46.98 | 77.63 | 32.44 | 14.81 | 38.12 | 38.35 | 72.74 | 83.95 |
| **55** | 11.54 | 81.07 | 83.39 | 87.01 | 42.85 | 45.92 | 77.57 | 32.20 | 14.82 | 38.00 | 38.36 | 72.74 | 83.90 |
| **56** | 11.57 | 81.75 | 84.02 | 87.90 | 40.69 | 43.93 | 74.90 | 30.87 | 14.65 | 38.03 | 39.07 | 72.84 | 83.43 |
| **57** | 11.53 | 80.95 | 83.32 | 87.24 | 37.58 | 38.50 | 68.36 | 26.90 | 13.92 | 37.86 | 40.57 | 67.81 | 80.59 |
| **58** | 11.55 | 81.69 | 84.16 | 88.12 | 40.82 | 44.94 | 76.13 | 32.38 | 14.79 | 38.64 | 38.15 | 73.33 | 83.80 |
| **59** | 11.56 | 82.00 | 84.23 | 88.12 | 37.39 | 40.57 | 69.82 | 27.14 | 14.24 | 37.22 | 41.95 | 68.22 | 81.45 |
| **60** | 11.56 | 82.81 | 85.08 | 88.93 | 40.42 | 38.55 | 72.31 | 27.65 | 14.21 | 36.64 | 41.23 | 69.43 | 81.56 |
| **61** | 11.58 | 81.57 | 83.81 | 87.46 | 38.25 | 41.15 | 71.10 | 29.15 | 14.42 | 38.57 | 40.30 | 70.67 | 82.59 |
| **62** | 11.58 | 82.06 | 84.30 | 88.12 | 38.31 | 37.97 | 68.95 | 27.17 | 14.10 | 37.85 | 41.64 | 69.01 | 81.30 |
| **63** | 11.56 | 81.63 | 83.88 | 87.97 | 35.13 | 32.07 | 62.67 | 22.38 | 13.26 | 35.63 | 45.49 | 64.52 | 76.62 |
| **64** | 11.59 | 81.50 | 83.81 | 87.46 | 34.57 | 33.56 | 62.26 | 22.52 | 13.29 | 36.06 | 45.73 | 62.89 | 75.76 |
| **65** | 11.54 | 81.45 | 84.15 | 87.80 | 37.36 | 41.79 | 72.68 | 27.62 | 14.17 | 36.44 | 41.92 | 69.37 | 81.19 |
| **66** | 11.59 | 81.70 | 84.15 | 88.32 | 42.01 | 43.25 | 74.65 | 29.13 | 14.34 | 37.04 | 40.36 | 69.90 | 82.84 |
| **67** | 11.57 | 81.57 | 84.08 | 87.80 | 38.58 | 35.91 | 69.01 | 26.40 | 14.10 | 36.90 | 42.32 | 68.36 | 80.18 |
| **68** | 11.55 | 80.57 | 82.82 | 86.87 | 39.86 | 40.32 | 73.26 | 29.09 | 14.53 | 37.32 | 40.05 | 70.57 | 82.18 |
| **69** | 11.52 | 80.39 | 82.68 | 86.42 | 38.38 | 39.79 | 70.33 | 26.85 | 14.13 | 36.83 | 40.86 | 69.31 | 80.45 |
| **70** | 11.53 | 81.07 | 83.39 | 87.31 | 37.85 | 37.97 | 68.64 | 26.15 | 14.00 | 36.98 | 41.07 | 69.43 | 80.08 |
| **71** | 11.57 | 80.82 | 83.24 | 86.87 | 39.80 | 42.01 | 73.16 | 28.61 | 14.34 | 36.70 | 40.97 | 71.14 | 81.62 |
| **72** | 11.53 | 80.70 | 82.75 | 86.57 | 38.63 | 41.81 | 71.66 | 27.70 | 14.28 | 36.89 | 41.48 | 68.50 | 80.85 |
| **73** | 11.52 | 81.26 | 83.46 | 87.09 | 40.57 | 44.84 | 75.30 | 31.42 | 14.67 | 38.32 | 38.76 | 72.74 | 83.40 |
| **74** | 11.51 | 80.76 | 82.89 | 86.79 | 41.32 | 43.42 | 74.98 | 28.87 | 14.31 | 36.60 | 40.43 | 70.54 | 81.71 |
| **75** | 11.52 | 81.19 | 83.53 | 87.60 | 42.30 | 44.11 | 76.49 | 28.89 | 14.20 | 36.18 | 39.95 | 69.86 | 81.45 |
| **76** | 11.52 | 80.08 | 82.33 | 86.20 | 40.49 | 44.28 | 74.98 | 30.70 | 14.67 | 37.95 | 39.24 | 72.19 | 82.96 |
| **77** | 11.54 | 81.19 | 83.60 | 87.38 | 42.26 | 41.48 | 74.20 | 29.41 | 14.34 | 37.27 | 39.70 | 70.54 | 83.05 |
| **78** | 11.54 | 81.44 | 84.02 | 87.60 | 42.44 | 44.11 | 76.27 | 30.96 | 14.59 | 37.55 | 39.11 | 73.33 | 83.33 |
| **79** | 11.56 | 82.19 | 84.51 | 88.49 | 36.77 | 36.76 | 67.89 | 23.33 | 13.56 | 34.64 | 43.66 | 65.08 | 76.44 |
| **80** | 11.54 | 81.32 | 83.53 | 87.38 | 38.47 | 34.49 | 67.99 | 25.85 | 13.61 | 36.98 | 42.91 | 67.67 | 79.05 |
| **81** | 11.54 | 80.76 | 82.82 | 86.79 | 41.64 | 46.18 | 76.32 | 31.85 | 14.85 | 38.25 | 38.46 | 73.20 | 83.71 |
| **82** | 11.57 | 82.63 | 85.21 | 88.84 | 36.99 | 40.35 | 69.75 | 27.12 | 14.18 | 37.19 | 42.05 | 68.56 | 80.39 |
| **83** | 11.56 | 82.81 | 85.42 | 89.20 | 36.72 | 32.80 | 65.76 | 21.36 | 13.08 | 33.30 | 44.85 | 63.88 | 74.87 |
| **84** | 11.56 | 82.50 | 84.93 | 88.98 | 37.47 | 37.65 | 68.41 | 27.32 | 14.10 | 38.20 | 41.74 | 68.95 | 80.18 |
| **85** | 11.57 | 83.18 | 85.71 | 89.74 | 36.92 | 38.91 | 68.79 | 24.54 | 14.00 | 35.41 | 43.03 | 66.29 | 78.44 |
| **86** | 11.54 | 82.13 | 84.37 | 88.42 | 38.40 | 37.04 | 68.76 | 26.19 | 14.09 | 36.92 | 42.44 | 68.05 | 79.69 |
| **87** | 11.57 | 83.00 | 85.57 | 89.45 | 38.89 | 34.44 | 67.47 | 23.90 | 13.70 | 35.16 | 43.32 | 65.93 | 77.96 |
| **88** | 11.53 | 82.44 | 85.08 | 89.30 | 36.74 | 38.98 | 69.17 | 26.68 | 14.34 | 37.15 | 41.99 | 67.74 | 81.32 |
| **89** | 11.56 | 81.82 | 84.51 | 88.56 | 40.46 | 43.27 | 73.18 | 28.88 | 14.36 | 37.05 | 41.17 | 70.40 | 81.44 |
| **90** | 11.58 | 81.69 | 84.23 | 88.27 | 35.48 | 34.87 | 66.04 | 24.86 | 13.65 | 35.98 | 44.65 | 65.76 | 76.18 |
| **91** | 11.52 | 81.75 | 84.23 | 88.12 | 35.08 | 32.40 | 65.26 | 22.74 | 13.43 | 34.76 | 44.88 | 64.95 | 74.93 |
| **92** | 11.50 | 81.38 | 83.67 | 87.46 | 38.20 | 42.82 | 71.74 | 28.57 | 14.27 | 37.36 | 41.37 | 70.71 | 81.20 |
| **93** | 11.52 | 80.95 | 83.24 | 87.16 | 40.09 | 43.75 | 73.97 | 29.91 | 14.37 | 37.65 | 40.33 | 70.93 | 83.01 |
| **94** | 11.54 | 82.44 | 85.01 | 89.01 | 38.71 | 41.68 | 71.59 | 28.39 | 14.21 | 37.53 | 40.07 | 69.86 | 82.02 |
| **95** | 11.55 | 82.13 | 84.65 | 88.71 | 37.30 | 34.09 | 64.85 | 24.02 | 13.65 | 36.80 | 43.32 | 66.29 | 78.17 |
| **96** | 11.57 | 82.44 | 84.79 | 88.49 | 37.52 | 36.51 | 67.15 | 23.85 | 13.53 | 35.28 | 43.99 | 65.45 | 78.54 |
| **97** | 11.50 | 81.75 | 83.95 | 87.75 | 36.81 | 36.06 | 67.01 | 23.43 | 13.61 | 34.98 | 43.66 | 65.72 | 77.92 |
| **98** | 11.53 | 82.88 | 85.49 | 89.43 | 36.12 | 35.25 | 66.09 | 23.47 | 13.59 | 35.41 | 44.02 | 65.80 | 77.53 |
| **99** | 11.54 | 82.25 | 84.71 | 88.47 | 34.55 | 37.35 | 65.19 | 25.65 | 13.69 | 38.26 | 42.88 | 67.68 | 79.15 |
| **100** | 11.54 | 82.00 | 84.44 | 88.34 | 34.89 | 34.57 | 63.78 | 23.06 | 13.62 | 36.11 | 44.95 | 64.70 | 77.49 |
| **101** | 11.54 | 82.31 | 84.93 | 88.86 | 35.30 | 34.59 | 63.84 | 21.40 | 13.24 | 34.20 | 45.76 | 62.60 | 74.49 |
| **102** | 11.55 | 82.19 | 84.72 | 88.71 | 35.68 | 34.14 | 64.99 | 23.18 | 13.44 | 35.14 | 45.25 | 64.10 | 76.12 |
| **103** | 11.55 | 82.44 | 84.93 | 89.01 | 36.57 | 37.47 | 68.82 | 25.79 | 13.86 | 35.71 | 43.56 | 67.63 | 77.80 |
| **104** | 11.56 | 82.37 | 85.08 | 88.79 | 34.40 | 36.31 | 64.33 | 25.36 | 13.61 | 37.24 | 44.02 | 66.99 | 78.15 |
| **105** | 11.57 | 82.87 | 85.50 | 89.38 | 35.97 | 34.09 | 66.57 | 23.46 | 13.67 | 35.13 | 44.51 | 65.19 | 77.50 |
| **106** | 11.55 | 83.06 | 85.43 | 89.38 | 34.28 | 33.89 | 63.99 | 21.30 | 13.33 | 33.88 | 45.48 | 63.27 | 75.01 |
| **107** | 11.55 | 82.69 | 85.08 | 88.93 | 34.48 | 33.61 | 62.04 | 21.33 | 13.56 | 35.59 | 45.47 | 63.54 | 75.44 |
| **108** | 11.58 | 82.50 | 84.93 | 88.56 | 35.59 | 36.28 | 65.72 | 24.31 | 13.64 | 35.98 | 44.54 | 65.17 | 76.99 |
| **109** | 11.55 | 82.87 | 85.57 | 89.60 | 36.17 | 38.30 | 68.48 | 25.54 | 14.12 | 36.12 | 43.14 | 66.36 | 78.62 |
| **110** | 11.56 | 81.57 | 83.95 | 87.75 | 36.44 | 35.30 | 67.39 | 24.29 | 14.12 | 35.60 | 43.35 | 65.19 | 78.30 |
| **111** | 11.57 | 81.88 | 84.30 | 87.97 | 40.17 | 42.52 | 73.26 | 29.43 | 14.28 | 37.43 | 41.03 | 70.71 | 81.94 |
| **112** | 11.53 | 80.76 | 83.10 | 87.16 | 36.28 | 36.51 | 67.45 | 23.48 | 13.78 | 34.74 | 44.46 | 65.72 | 77.52 |
| **113** | 11.51 | 82.01 | 84.43 | 88.10 | 37.41 | 35.48 | 68.02 | 22.95 | 13.73 | 34.24 | 43.88 | 65.12 | 76.97 |
| **114** | 11.55 | 81.14 | 83.59 | 87.21 | 37.05 | 37.40 | 68.10 | 23.84 | 13.54 | 35.07 | 43.49 | 65.12 | 78.16 |
| **115** | 11.57 | 82.13 | 84.64 | 88.47 | 36.41 | 39.64 | 69.28 | 25.86 | 14.05 | 36.33 | 42.29 | 67.03 | 79.76 |
| **116** | 11.54 | 81.44 | 84.09 | 87.68 | 37.37 | 42.04 | 71.09 | 26.80 | 13.90 | 36.27 | 41.90 | 67.94 | 80.06 |
| **117** | 11.53 | 81.07 | 83.24 | 87.09 | 38.76 | 38.93 | 70.84 | 27.71 | 14.37 | 37.23 | 41.48 | 70.02 | 81.30 |
| **118** | 11.55 | 81.50 | 84.02 | 88.12 | 34.91 | 39.16 | 67.09 | 25.39 | 13.76 | 37.02 | 43.46 | 67.24 | 78.36 |
| **119** | 11.56 | 80.70 | 82.96 | 86.57 | 36.08 | 39.03 | 68.52 | 25.38 | 14.14 | 36.02 | 43.53 | 66.95 | 78.11 |
| **120** | 11.55 | 81.94 | 84.51 | 88.42 | 37.85 | 36.53 | 67.47 | 25.16 | 14.10 | 36.52 | 42.76 | 66.29 | 79.30 |
| **121** | 11.56 | 81.57 | 84.02 | 87.90 | 36.99 | 38.45 | 67.62 | 25.73 | 13.84 | 36.84 | 42.76 | 66.95 | 77.54 |
| **122** | 11.57 | 81.88 | 84.30 | 88.34 | 34.89 | 34.42 | 63.15 | 22.97 | 13.89 | 36.59 | 43.58 | 63.80 | 77.28 |
| **123** | 11.54 | 81.69 | 84.23 | 88.12 | 36.90 | 38.68 | 69.93 | 26.05 | 14.26 | 35.99 | 42.58 | 67.30 | 77.58 |
| **124** | 11.58 | 82.87 | 85.57 | 89.30 | 36.65 | 41.56 | 70.03 | 28.51 | 14.09 | 37.51 | 41.95 | 68.92 | 80.08 |
| **125** | 11.54 | 81.82 | 84.09 | 87.68 | 35.08 | 31.34 | 62.56 | 20.65 | 13.17 | 33.72 | 46.14 | 62.07 | 73.67 |
| **126** | 11.57 | 81.57 | 84.16 | 88.12 | 39.93 | 40.62 | 71.55 | 26.12 | 14.19 | 35.79 | 42.11 | 67.67 | 80.30 |
| **127** | 11.56 | 81.07 | 83.39 | 87.31 | 41.04 | 44.16 | 74.60 | 29.36 | 14.50 | 36.86 | 40.63 | 70.40 | 82.55 |
| **128** | 11.59 | 81.63 | 84.02 | 87.97 | 38.43 | 40.14 | 69.30 | 26.25 | 13.84 | 35.73 | 43.63 | 66.99 | 77.87 |
| **129** | 11.57 | 81.63 | 83.73 | 87.36 | 34.84 | 37.65 | 65.99 | 24.85 | 13.76 | 36.28 | 44.10 | 66.01 | 78.36 |
| **130** | 11.57 | 81.76 | 83.94 | 87.73 | 36.64 | 36.41 | 69.22 | 24.82 | 13.79 | 35.19 | 43.66 | 65.76 | 78.98 |
| **131** | 11.55 | 82.00 | 84.37 | 88.19 | 40.07 | 41.03 | 71.93 | 28.51 | 14.45 | 37.53 | 40.04 | 69.73 | 81.76 |
| **132** | 11.57 | 81.69 | 84.30 | 87.97 | 42.10 | 45.87 | 76.63 | 31.67 | 14.80 | 37.97 | 38.84 | 73.13 | 83.55 |
| **133** | 11.55 | 81.94 | 84.44 | 88.34 | 35.26 | 33.78 | 63.31 | 21.84 | 13.55 | 35.22 | 45.00 | 63.27 | 75.44 |
| **134** | 11.55 | 82.56 | 85.15 | 88.86 | 38.25 | 40.09 | 69.55 | 27.34 | 13.99 | 37.43 | 41.80 | 68.96 | 80.65 |
| **135** | 11.52 | 81.19 | 83.39 | 87.16 | 37.30 | 37.97 | 68.35 | 25.70 | 14.12 | 36.49 | 42.48 | 66.87 | 79.06 |
| **136** | 11.51 | 82.13 | 84.72 | 88.64 | 40.57 | 40.80 | 72.01 | 29.11 | 14.40 | 37.81 | 40.73 | 70.02 | 81.48 |
| **137** | 11.54 | 81.63 | 84.09 | 87.83 | 34.40 | 31.74 | 62.49 | 20.28 | 13.08 | 33.28 | 46.93 | 61.65 | 73.10 |
| **138** | 11.53 | 82.06 | 84.65 | 88.71 | 35.04 | 35.02 | 63.69 | 23.17 | 13.20 | 35.66 | 45.48 | 64.10 | 75.92 |
| **139** | 11.51 | 81.44 | 83.74 | 87.53 | 34.35 | 32.55 | 63.21 | 21.08 | 13.28 | 34.07 | 46.22 | 62.60 | 74.24 |
| **140** | 11.54 | 81.26 | 83.53 | 87.31 | 39.36 | 38.73 | 71.28 | 26.42 | 14.21 | 35.88 | 41.09 | 68.22 | 79.61 |
| **141** | 11.52 | 81.32 | 83.74 | 87.38 | 39.87 | 39.61 | 71.15 | 26.51 | 13.83 | 36.11 | 42.46 | 67.64 | 80.23 |
| **142** | 11.51 | 81.38 | 83.74 | 87.75 | 40.06 | 43.65 | 73.60 | 29.65 | 14.48 | 37.33 | 40.39 | 70.46 | 81.45 |
| **143** | 11.55 | 80.88 | 83.32 | 87.24 | 39.86 | 44.03 | 71.36 | 29.36 | 14.46 | 38.51 | 39.81 | 70.67 | 82.52 |
| **144** | 11.55 | 81.94 | 84.15 | 88.17 | 36.24 | 39.57 | 67.55 | 25.87 | 14.04 | 37.28 | 42.71 | 66.86 | 79.12 |
| **145** | 11.55 | 81.01 | 83.66 | 87.51 | 36.15 | 37.19 | 66.56 | 23.00 | 13.75 | 34.81 | 43.99 | 64.19 | 77.13 |
| **146** | 11.52 | 82.32 | 84.64 | 88.54 | 38.51 | 40.50 | 70.37 | 27.52 | 14.15 | 37.36 | 41.77 | 68.36 | 81.08 |
| **147** | 11.56 | 81.57 | 84.16 | 88.19 | 36.13 | 34.84 | 66.76 | 22.89 | 13.80 | 34.63 | 44.43 | 64.37 | 77.61 |
| **148** | 11.56 | 82.62 | 85.22 | 89.15 | 37.76 | 39.61 | 68.50 | 26.49 | 13.92 | 37.17 | 42.39 | 66.51 | 79.64 |
| **149** | 11.55 | 82.44 | 85.01 | 89.15 | 34.17 | 32.24 | 63.10 | 20.87 | 13.08 | 33.67 | 46.66 | 62.07 | 73.35 |
| **150** | 11.58 | 82.75 | 85.43 | 88.79 | 33.58 | 30.76 | 60.75 | 20.30 | 13.08 | 33.98 | 46.66 | 61.65 | 73.45 |
| **151** | 11.59 | 83.12 | 85.78 | 89.67 | 34.47 | 33.41 | 63.29 | 21.87 | 13.29 | 34.56 | 45.63 | 63.57 | 75.42 |
| **152** | 11.55 | 82.87 | 85.85 | 89.89 | 36.77 | 35.02 | 67.41 | 23.51 | 13.50 | 34.68 | 44.35 | 65.23 | 77.48 |
| **153** | 11.53 | 81.94 | 84.37 | 88.42 | 36.12 | 35.96 | 67.82 | 22.47 | 13.32 | 33.72 | 44.83 | 64.51 | 76.67 |
| **154** | 11.54 | 82.25 | 85.01 | 88.93 | 35.42 | 35.02 | 66.32 | 21.33 | 13.16 | 33.23 | 45.34 | 63.27 | 75.24 |
| **155** | 11.55 | 82.06 | 84.58 | 88.42 | 34.29 | 39.01 | 67.75 | 25.70 | 13.86 | 36.08 | 43.71 | 66.27 | 78.14 |
| **156** | 11.54 | 82.19 | 84.58 | 88.56 | 34.26 | 31.16 | 61.69 | 20.65 | 12.95 | 34.03 | 46.49 | 62.18 | 74.16 |
| **157** | 11.55 | 82.37 | 84.79 | 88.79 | 34.82 | 29.19 | 62.48 | 20.86 | 13.25 | 33.96 | 46.57 | 61.85 | 74.82 |
| **158** | 11.56 | 82.37 | 84.86 | 88.93 | 34.64 | 32.07 | 62.70 | 22.11 | 13.29 | 35.23 | 44.63 | 63.08 | 75.00 |
| **159** | 11.56 | 82.69 | 85.28 | 89.13 | 35.08 | 35.91 | 66.57 | 25.66 | 13.86 | 36.36 | 43.35 | 67.03 | 78.00 |
| **160** | 11.55 | 82.88 | 85.49 | 89.43 | 38.20 | 40.70 | 70.16 | 27.80 | 13.98 | 37.33 | 42.26 | 69.21 | 80.13 |
| **161** | 11.54 | 82.13 | 84.71 | 88.69 | 35.57 | 39.97 | 70.17 | 26.72 | 14.00 | 36.03 | 43.18 | 67.44 | 79.32 |
| **162** | 11.55 | 82.44 | 84.86 | 88.93 | 34.93 | 37.17 | 66.13 | 24.80 | 13.70 | 36.16 | 44.42 | 65.33 | 77.74 |
| **163** | 11.55 | 82.56 | 85.08 | 88.93 | 34.80 | 35.17 | 66.19 | 24.98 | 13.61 | 36.21 | 43.95 | 65.52 | 77.35 |
| **164** | 11.55 | 82.25 | 84.72 | 88.86 | 36.74 | 39.61 | 68.69 | 25.85 | 13.93 | 35.87 | 43.18 | 66.46 | 78.97 |
| **165** | 11.55 | 82.06 | 84.51 | 88.56 | 34.40 | 34.64 | 63.91 | 22.21 | 13.47 | 34.85 | 45.18 | 64.10 | 75.52 |
| **166** | 11.56 | 82.87 | 85.50 | 89.60 | 36.85 | 34.47 | 65.93 | 23.28 | 13.26 | 34.77 | 44.95 | 64.10 | 75.92 |
| **167** | 11.54 | 82.50 | 85.01 | 89.01 | 35.10 | 34.54 | 65.43 | 23.15 | 13.52 | 34.88 | 45.12 | 64.10 | 74.60 |
| **168** | 11.57 | 82.50 | 84.93 | 88.71 | 38.11 | 42.39 | 72.32 | 27.90 | 14.21 | 36.28 | 41.75 | 69.75 | 80.44 |
| **169** | 11.56 | 82.87 | 85.43 | 89.45 | 37.05 | 40.47 | 69.13 | 27.41 | 14.11 | 37.16 | 42.62 | 69.12 | 80.10 |
| **170** | 11.55 | 82.00 | 84.37 | 88.27 | 34.64 | 36.23 | 66.14 | 25.88 | 13.73 | 36.74 | 43.97 | 66.99 | 77.47 |
| **171** | 11.55 | 81.82 | 84.30 | 88.19 | 40.37 | 41.00 | 72.35 | 28.13 | 14.38 | 37.00 | 41.41 | 68.05 | 82.06 |
| **172** | 11.52 | 81.01 | 83.53 | 87.46 | 40.53 | 40.45 | 71.48 | 28.41 | 14.18 | 37.61 | 40.51 | 69.43 | 81.74 |
| **173** | 11.50 | 81.63 | 83.88 | 87.46 | 38.43 | 41.43 | 71.43 | 27.75 | 14.27 | 36.96 | 41.81 | 69.73 | 81.84 |
| **174** | 11.53 | 81.13 | 83.74 | 87.53 | 37.14 | 38.40 | 67.97 | 26.63 | 14.09 | 37.82 | 42.18 | 68.20 | 80.20 |
| **175** | 11.56 | 82.31 | 84.93 | 88.71 | 39.98 | 41.99 | 72.42 | 29.17 | 14.56 | 37.65 | 40.64 | 70.33 | 81.87 |
| **176** | 11.54 | 80.95 | 83.31 | 87.07 | 36.26 | 32.58 | 64.92 | 24.12 | 13.79 | 36.80 | 43.80 | 65.76 | 78.20 |
| **177** | 11.53 | 82.57 | 85.14 | 89.20 | 35.73 | 39.16 | 68.74 | 27.06 | 14.25 | 37.25 | 42.04 | 67.93 | 80.38 |
| **178** | 11.55 | 82.06 | 84.58 | 88.56 | 39.20 | 41.48 | 71.52 | 28.48 | 14.36 | 37.42 | 41.09 | 68.47 | 81.02 |
| **179** | 11.51 | 81.69 | 84.51 | 88.42 | 37.54 | 42.14 | 71.42 | 27.89 | 14.23 | 36.74 | 41.54 | 69.17 | 80.81 |
| **180** | 11.55 | 81.69 | 84.30 | 88.34 | 35.59 | 36.26 | 66.47 | 26.18 | 13.98 | 36.96 | 43.39 | 66.99 | 78.40 |
| **181** | 11.56 | 83.25 | 85.78 | 89.82 | 38.94 | 40.25 | 72.52 | 29.20 | 14.58 | 37.79 | 40.71 | 70.73 | 82.34 |
| **182** | 11.54 | 82.50 | 85.08 | 89.23 | 36.97 | 37.65 | 67.30 | 25.75 | 13.99 | 37.20 | 42.61 | 68.32 | 78.89 |
| **183** | 11.56 | 81.38 | 83.67 | 87.75 | 36.46 | 38.73 | 67.44 | 26.96 | 14.11 | 38.42 | 42.00 | 68.90 | 80.38 |
| **184** | 11.58 | 81.63 | 84.09 | 88.19 | 40.39 | 41.26 | 71.12 | 28.89 | 14.23 | 38.18 | 39.77 | 70.51 | 82.28 |
| **185** | 11.57 | 81.57 | 84.09 | 88.12 | 41.86 | 45.97 | 76.82 | 31.97 | 14.81 | 38.03 | 38.70 | 73.20 | 83.59 |
| **186** | 11.57 | 83.00 | 85.64 | 89.82 | 38.63 | 41.86 | 70.93 | 28.58 | 14.11 | 37.51 | 41.39 | 69.12 | 80.50 |
| **187** | 11.54 | 81.69 | 84.02 | 87.90 | 37.98 | 40.52 | 69.56 | 27.61 | 14.13 | 37.16 | 42.11 | 69.18 | 79.82 |
